# Superlattice Engineering in Relaxor Ferroelectric-based Capacitors for Ultra-Highly Efficient Recoverable Energy Storage Density

**DOI:** 10.1038/s41467-026-77971-6

**Published:** 2026-10-05

**Authors:** Matilde M. G. S. Oliveira, Ampattu R. Jayakrishnan, Duarte J. M. Ribeiro, Tiago Rodrigues, Jingye Zou, Surya K. P. Nair, Raluca Negrea, Marian C. Istrate, Manuel J. L. F. Rodrigues, Michael Belsley, Bernardo G. Almeida, Mario Pereira, Judith L. MacManus-Driscoll, Ignasi Fina, Florencio Sánchez, Luís Marques, José P. B. Silva

**Affiliations:** 1 Physics Center of Minho and Porto Universities (CF-UM-UP), University of Minho, Campus de Gualtar, 4710-057 Braga, Portugal; 2 Laboratory of Physics for Materials and Emergent Technologies, LapMET, University of Minho, 4710-057 Braga, Portugal; 3 Institut de Ciència de Materials de Barcelona (ICMAB-CSIC), Carrer dels Til.lers, 08193 Cerdanyola del Vallès Barcelona, Spain; 4 Brunel Centre for Advanced Solidification Technology (BCAST), Brunel University London, Uxbridge, Middlesex UB8 3PH UK; 5 National Institute of Materials Physics, 405A Atomistilor, 077125 Magurele, Romania; 6 Department of Materials Science & Metallurgy, University of Cambridge, 27 Charles Babbage Road, Cambridge, CB3 0FS UK

**Keywords:** Ferroelectrics and multiferroics, Surfaces, interfaces and thin films

## Abstract

In this work, we show that superlattice engineering, through the combination of relaxor ferroelectric 0.5Ba(Zr_0.2_Ti_0.8_)O_3_−0.5(Ba_0.7_Ca_0.3_)TiO_3_ and paraelectric SrTiO_3_ layers, is a powerful approach to simultaneously optimize energy density and efficiency of recoverable energy storage at low applied electric fields, outperforming both single layer 0.5Ba(Zr_0.2_Ti_0.8_)O_3_−0.5(Ba_0.7_Ca_0.3_)TiO_3_ thin film and other lead-free ferroelectric and relaxor ferroelectric thin film capacitors. The mechanisms responsible are a) decreasing the size of polar nanoregions which promote relaxor behavior by superlattice confinement and b) formation of a strain gradient in the superlattice which plays a significant role in modification of the built-in electric field, to enhance imprint, allowing for further enhancement of the energy storage performance. Overall, our interface-engineered Pb-free 0.5Ba(Zr_0.2_Ti_0.8_)O_3_−0.5(Ba_0.7_Ca_0.3_)TiO_3_/SrTiO_3_ superlattices show record dielectric energy storage efficiency close to 99% at 920 kV/cm, with high recoverable energy storage density of 12.6 J/cm^3^, significantly outperforming other lead-free ferroelectric and relaxor ferroelectric thin film capacitors. Our thin film system meets the performance requirements for environmentally friendly energy storage devices for future self-powered electronics.

## Abstract (web summary)

Superlattice engineering, combining relaxor ferroelectric and paraelectric layers, provides a promising strategy to simultaneously enhance energy storage efficiency and recoverable energy density at low applied electric fields.

## Introduction

Superlattices (SLs), composed of periodic nanometer-scale structures of alternating thin layers of perovskite ferroelectric (FE) and paraelectric (PE) materials, have attracted increasing attention because they can support polarization states that are not accessible in bulk form^1,2^. When FE and PE thin layers are alternated, the periodic interfaces impose electrostatic and mechanical constraints that interfere with the formation of macro-domains. Instead of these larger regions with uniform polarization, more complex configurations may appear. For example, polar vortices, spirals or flux-closure arrangements, where the polarization rotates continuously in space rather than switching within a domain wall^3^. These structures result from a balance between elastic strain and electrostatic coupling, and their stability depends on the SL periodicity and individual layer thickness. In this situation, polarization is no longer long-range defined in the usual sense but becomes spatially modulated and confined^4^.

In FE/PE SLs, rotational polarization textures have been observed due to the inability of the system to sustain macro-domains^5^. Polarization tends to fragment into nanoscale regions with continuously varying orientation. The precise layering of dissimilar materials (e.g., ferroelectric and paraelectric) introduces strain, interfaces, and artificial disorder that emulate the chemical heterogeneity found in traditional relaxor ferroelectric materials (RFEs) that exhibit polar nanoregions (PNRs)^1,6^.

While FE/PE SLs have been intensively investigated for next-generation electronics, their practical implementation for energy storage (ES) applications have been scarcely investigated. For example, Aramberri et al. theoretically demonstrated that PbTiO_3_/SrTiO_3_ SLs can be engineered as artificial antiferroelectrics to optimize recoverable energy storage density (ESD) and efficiency (ŋ)^2^. These SLs exhibit polarization vortex structures and strain-dependent hysteresis behavior, which directly controls their ES performance. A recoverable ESD of ~6 J/cm^3^ at 550 kV/cm was then achieved experimentally in PbTiO_3_/SrTiO_3_ SLs^7^.

Phase field simulations demonstrated the relevance of BaTiO_3_/SrTiO_3_ and BiFeO_3_/SrTiO_3_ SLs where strain engineering is important to tune the polar topologies and enhance the ES performance^8,9^. For instance, Y. Liu et al. demonstrated that vortex, spiral, and in-plane polar structures can be stabilized in BiFeO_3_/SrTiO_3_ multilayers by tailoring the strain state and layer thickness^8^. The emergence of polar vortex topologies can enhance the ES performance, since the remnant polarization in vortex topologies can be close to zero.

On the other hand, the combination of RFE and PE layers in a multilayered device seems to be promising for ES applications, since the use of RFE instead of FE layers can already promote the formation of PNRs, which usually enhance the ES properties^10^. M. D. Nguyen et al. demonstrated that RFE/PE multilayered devices combining PE-like Ba_0.6_Sr_0.4_TiO_3_ with RFE BaZr_0.4_Ti_0.6_O_3_ (BZT) layers on SrTiO_3_-buffered Si substrates can exhibit a large ESD of ≈165.6 J/cm^3^, with an ŋ of 93 %, due to the extremely large breakdown field of 7.5 MV/cm^11^. In another work, X. Zhang et al. showed a recoverable ESD of 65.8 J/cm^3^ with an ŋ of 72.3% in a 6.7 nm-per-layer BiFeO_3_/SrTiO_3_ multilayer configuration^12^. In fact, perovskite RFEs and FEs for ES are usually investigated at high electric fields (>3 MV/cm)^1,13–17^. However, the use of large electric fields limits their practical applications, particularly when it comes to their integration in portable, wearable, and miniaturized pulse-power electronic systems, such as advanced capacitors, micro-electromechanical systems (MEMS), and sensors^18,19^. In the context of thin-film capacitors, we define low electric field operation as ≤ 2.5 MV/cm. Typically, >3 MV/cm fields are required to reach the highest reported ESD values in perovskite RFE/FE thin films. For a film thickness of 200 nm (as used here), this corresponds to an applied voltage of <50 V, consistent with a supply voltage for typical miniaturized/wearable electronics. For this voltage range, RFEs are used for high-density, ultra-fast ES in miniaturized electronics^18^. For instance, in wearable and implantable devices, space is the primary constraint, and for that reason, RFE thin films, instead of ceramic-based capacitors, are highly required.

Therefore, in this work we synergistically combined both strategies in order to achieve ultra-high ŋ at low electric fields. Thus, we investigated an RFE/PE SL approach. While phase-field simulations can be relevant to predict the existence of PNRs^20,21^, it is well known that 0.5Ba(Zr_0.2_Ti_0.8_)O_3_−0.5(Ba_0.7_Ca_0.3_)TiO_3_ (BCZT) thin films are RFEs that already have PNRs, as widely reported^22–26^. Therefore, BCZT was chosen because it has high maximum polarization and it can also exhibit RFE-like behavior and diffuse phase transitions when there is cation disorder on both A and B sites^22–26^. SrTiO_3_ (STO) was chosen as the PE barrier to stabilize or induce polar phases in the BCZT adjacent layers. Therefore, in these RFE/PE SL structures, we anticipate that spatial confinement prevents PNRs from growing larger.

Using our SL approach, we demonstrate that typical RFE behavior observed in a BCZT thin film capacitor can be significantly enhanced through SL engineering. This leads to an improvement of the ESD and ŋ of the capacitors by about 40%. We additionally observed an imprint effect that further enhances the ES performance due to a strain gradient in the SL that causes the development of a built-in internal field across the capacitors. Overall, we demonstrate an interface-induced polarization fragmentation approach using epitaxial SLs, which leads to record ŋ performance in lead-free RFEs. Our route can also be adopted more broadly to improve the ES properties of wide-ranging dielectric thin film capacitors.

## Results and Discussion

### High-quality epitaxial BCZT/STO superlattices

**Fig. 1:**
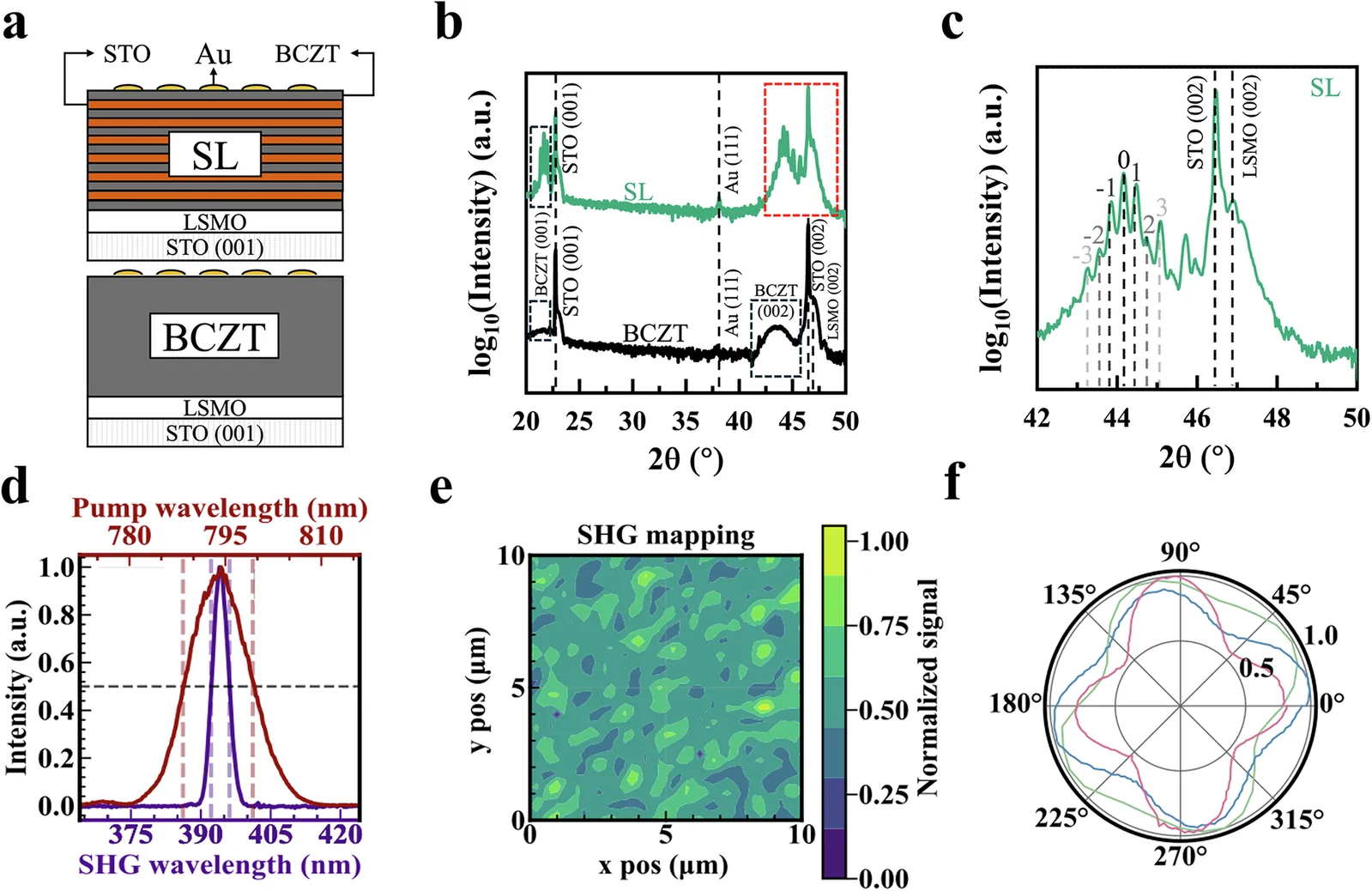
Structural and nonlinear optical characterization of the BCZT thin film and BCZT/STO SL. **a** Schematic representation of the capacitor structures of the BCZT film and the BCZT/STO SL grown on STO (001) substrates with La_0.7_Sr_0.3_MnO_3_ (LSMO) bottom electrodes and Au top electrodes. **b** XRD θ−2θ patterns of the BCZT film and BCZT/STO SL. **c** Enlarged view of the SL (002) reflection region highlighting the series of satellite peaks arising from the periodic BCZT/STO stacking. **d** Second-harmonic generation (SHG) spectrum of the BCZT/STO SL together with the fundamental pump spectrum. **e** SHG intensity map acquired over a 10 × 10 µm^2^ area, revealing spatial variations in the nonlinear response while maintaining a measurable SHG signal across the scanned region. **f** Polarization-resolved SHG (p-SHG) polar plots measured at different positions on the film, indicating variations in the effective nonlinear response due to local differences in ferroelectric domain configurations.

Figure 1a shows the stack structure of both thin film capacitors. The XRD patterns of the BCZT film and the BCZT/STO SL are shown in Fig. 1b. Apart from a very small peak attributed to the (111) Au from the top electrode, in both samples, the XRD peaks at 2θ ~ 23° and 2θ ~ 46° corresponding to the STO (001) and STO (002) reflection, with the corresponding LSMO peaks overlapped, are visible. In the case of the BCZT film, the XRD pattern reveals a single perovskite reflection located near the expected positions of the (001) and (002) peaks, 2θ ~ 22° and 2θ ~ 44°, respectively, confirming the textured growth of the BCZT layer.

In contrast, the diffraction pattern of the BCZT/STO SL shows a different behavior. Instead of a single BCZT peak, a series of additional reflections appears symmetrically around the main Bragg peak, forming a set of satellite peaks. A magnified view of the SL reflection region, marked in Fig. 1b, is shown in Fig. 1c, where multiple satellite peaks can be clearly seen. These satellite peaks originate from the periodic alternation of BCZT and STO layers and are characteristic of a high-quality SL structure^27,28^. The periodic spacing between these peaks is directly related to the periodicity of the SL through the Kiessig interference condition (Eq. 1)^29^. According to this relation, the SL periodicity Λ can be calculated from the angular separation between adjacent satellite peaks using:

1 $${\Lambda }_{{{\rm{SL}}}}=\frac{\lambda }{2\left[\sin \left({\theta }_{n+1}\right)-\sin \left({\theta }_{n}\right)\right]}$$

where λ is the X-ray wavelength and θ_n_ and θ_n+1_ are the diffraction angles of successive satellite peaks. The periodicity extracted using this method is consistent with the bilayer thickness of approximately 30 nm, corresponding to the combined thickness of the 15 nm BCZT and 15 nm STO layers in the SL architecture. Moreover, these XRD results confirm that both samples are single-phase and (00 l) oriented.

Second-harmonic generation (SHG) measurements were performed in the BCZT/STO SL to confirm its polar nature. SHG signal from the BCZT/STO SL is shown in Fig. 1d, consistent with polar contributions from the BCZT layers. To assess the lateral uniformity, we mapped the SHG intensity over a 10 × 10 µm^2^ area using 250 nm translation steps, well below the estimated ~1.3 µm excitation spot diameter (Fig. 1e). The resulting map shows SHG throughout the scanned region, modulated by local maxima and minima. In multidomain ferroelectrics, SHG can be locally suppressed by destructive interference when oppositely polarized domains contribute equally within the probed volume^30,31^. Here, the persistence of an appreciable SHG signal across the mapped area suggests that the probed regions generally retain a non-zero net polar contribution on a µm scale. The spatial modulation most likely reflects local variations in probed domain configurations. Polarization-resolved SHG (p-SHG) measurements further support this interpretation. When probing different locations separated on the micron scale, the p-SHG polar plots change (Fig. 1f), indicating that the effective nonlinear tensor sampled varies across the film, which is expected when multiple domains contribute to the signal and their relative populations and/or in-plane orientations vary. Finally, the spectra in Fig. 1d show that the detected SHG bandwidth (FWHM ≈ 3.8 nm) is substantially narrower than the fundamental bandwidth (FWHM ≈ 10.9 nm). This narrowing is expected for femtosecond SHG: for a near-Gaussian spectrum, the SHG field spectrum corresponds to a self-convolution of the fundamental, and when expressed in wavelength units, the SHG bandwidth is reduced by approximately √2/4~0.35× the fundamental bandwidth. Using the measured fundamental width, this predicts ~3.8 nm for the SHG width, in excellent agreement with the experiment. This agreement is a useful internal check that the recorded peak is dominated by true frequency-doubled light.

**Fig. 2:**
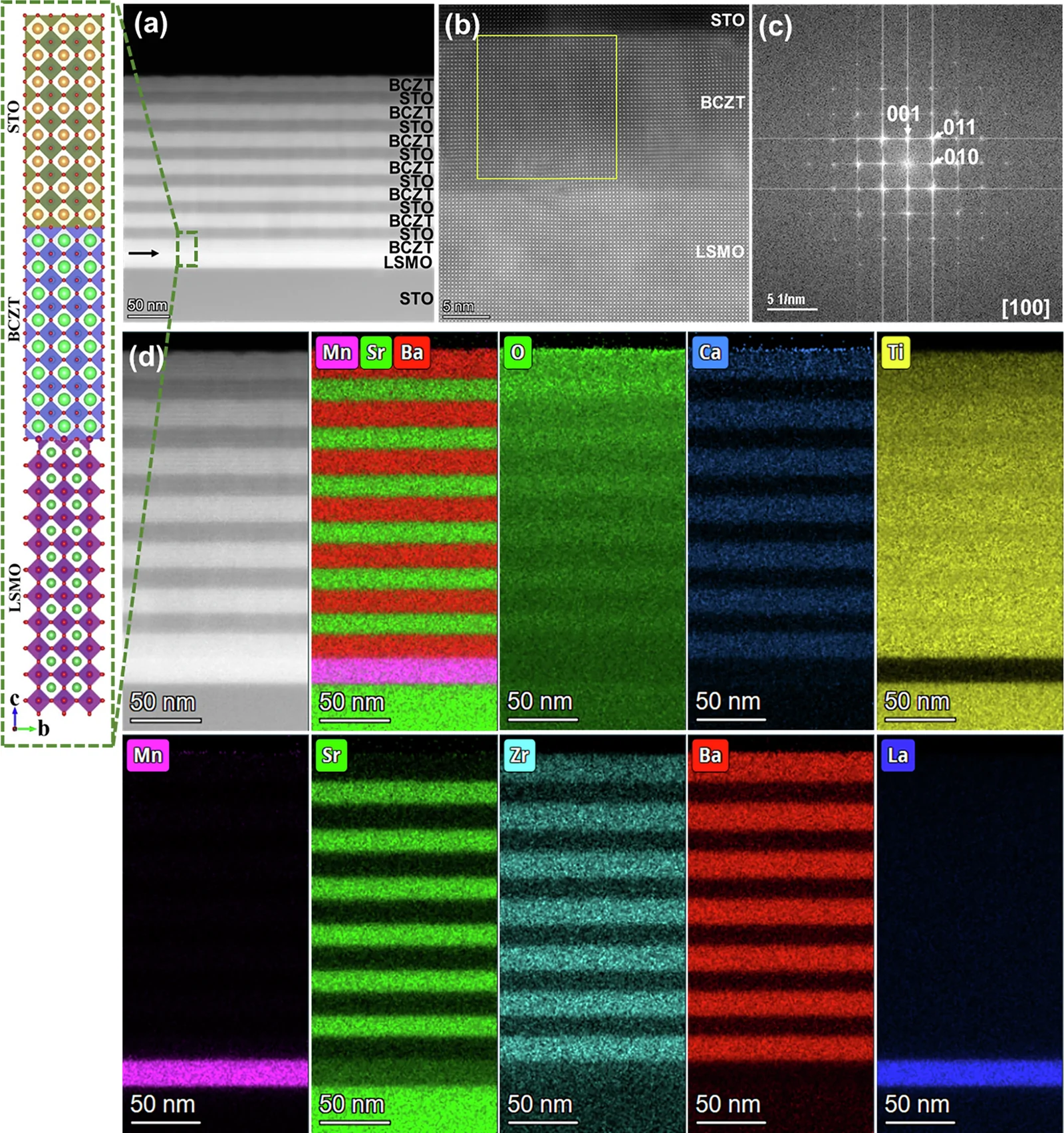
Overview of the STO-BCZT/LSMO/STO SL. **a** Cross-sectional STEM overview image showing the periodic stacking of alternating BCZT and STO layers grown on an LSMO-buffered STO substrate. Individual layers are labelled, highlighting the well-defined SL periodicity and sharp interfaces. The sketch on the left shows the ideal atomic structure in one period. **b** Atomic-resolution HAADF-STEM image across the SL, revealing coherent epitaxial growth of the BCZT and STO layers on LSMO. The yellow box marks the region used for Fourier analysis. **c** Fast Fourier transform (FFT) obtained from the selected region in (**b**), indexed along the [100] zone axis, confirming the perovskite structure and crystallographic alignment of the BCZT layers. **d** HAADF-STEM image and corresponding energy dispersive X-ray spectroscopy (EDS) elemental maps of the 7xSTO-BCZT/LSMO/STO SL.

To confirm the periodic architecture in the SL inferred from the XRD analysis, high-resolution scanning transmission electron microscopy (STEM) was performed. Figure 2a presents a structural and chemical analysis of the SL grown on an LSMO-buffered STO substrate. The schematic representation along with the cross-sectional high-angle annular dark-field (HAADF)-STEM overview image shown in Fig. 2a reveals a well-defined SL architecture consisting of alternating BCZT and STO layers deposited on top of the conductive LSMO bottom electrode. The individual layers are clearly distinguishable and exhibit uniform thickness throughout the stack, confirming successful fabrication of the designed SL. The interfaces are sharp and planar throughout the structure, with no evidence of layer waviness, thickness modulation, or macroscopic intermixing. This indicates a high degree of control during the growth process and confirms the formation of a structurally SL system with excellent preserved periodicity.

Higher magnification HAADF-STEM imaging, shown in Fig. 2b, provides insight into the atomic structure of the SL. The image shows continuous atomic columns extending across the STO, BCZT, and LSMO layers, which proves coherent epitaxial growth throughout the entire structure. Furthermore, the uniform contrast of each layer confirms the presence of abrupt interfaces with minimal cation interdiffusion, reflecting the high structural integrity of the SL.

The crystallographic quality of the BCZT layers is further confirmed by the fast Fourier transform (FFT) obtained from the highlighted region in Fig. 2b, shown in Fig. 2c. The FFT pattern is indexed along the [100] zone axis and displays sharp, well-defined reflections corresponding to the perovskite structure, including the (001), (010), and (011) reflections. The absence of additional reflections or diffuse scattering indicates that the BCZT layers retain a single-phase perovskite structure without the formation of secondary phases or significant structural disorder. The alignment of the diffraction spots confirms that the BCZT layers are epitaxially aligned with both the STO spacer layers and the LSMO electrode, forming a coherent heterostructure throughout the SL stack. The lattice parameters extracted from the FFT analysis are approximately a = b = 3.97 Å and c = 4.06 Å.

The chemical integrity of the SL was further examined using STEM-EDS elemental mapping, as shown in Fig. 2d. The elemental maps reveal a well-defined periodic chemical modulation consistent with the designed layer sequence. Mn and La are confined to the LSMO bottom electrode, while Sr is predominantly localized within the STO spacer layers. In contrast, Ba, Ca, and Zr are clearly enriched in the BCZT layers, confirming the compositional separation between the different constituents of the SL. The Ti and O signals remain continuous across the entire heterostructure, reflecting the common perovskite framework and confirming the absence of secondary phases or structural discontinuities. The sharp compositional transitions observed across the interfaces (shown in more detail in Fig. S1) demonstrate chemically abrupt layers and high compositional fidelity, with no measurable cation interdiffusion beyond the spatial resolution of the technique.

Overall, the combined HAADF-STEM and STEM-EDS analysis demonstrates that the BCZT/STO SL exhibits excellent structural and chemical quality, characterized by coherent epitaxy, sharp interfaces, and well-defined compositional modulation.

### Ferroelectric and dielectric properties

**Fig. 3:**
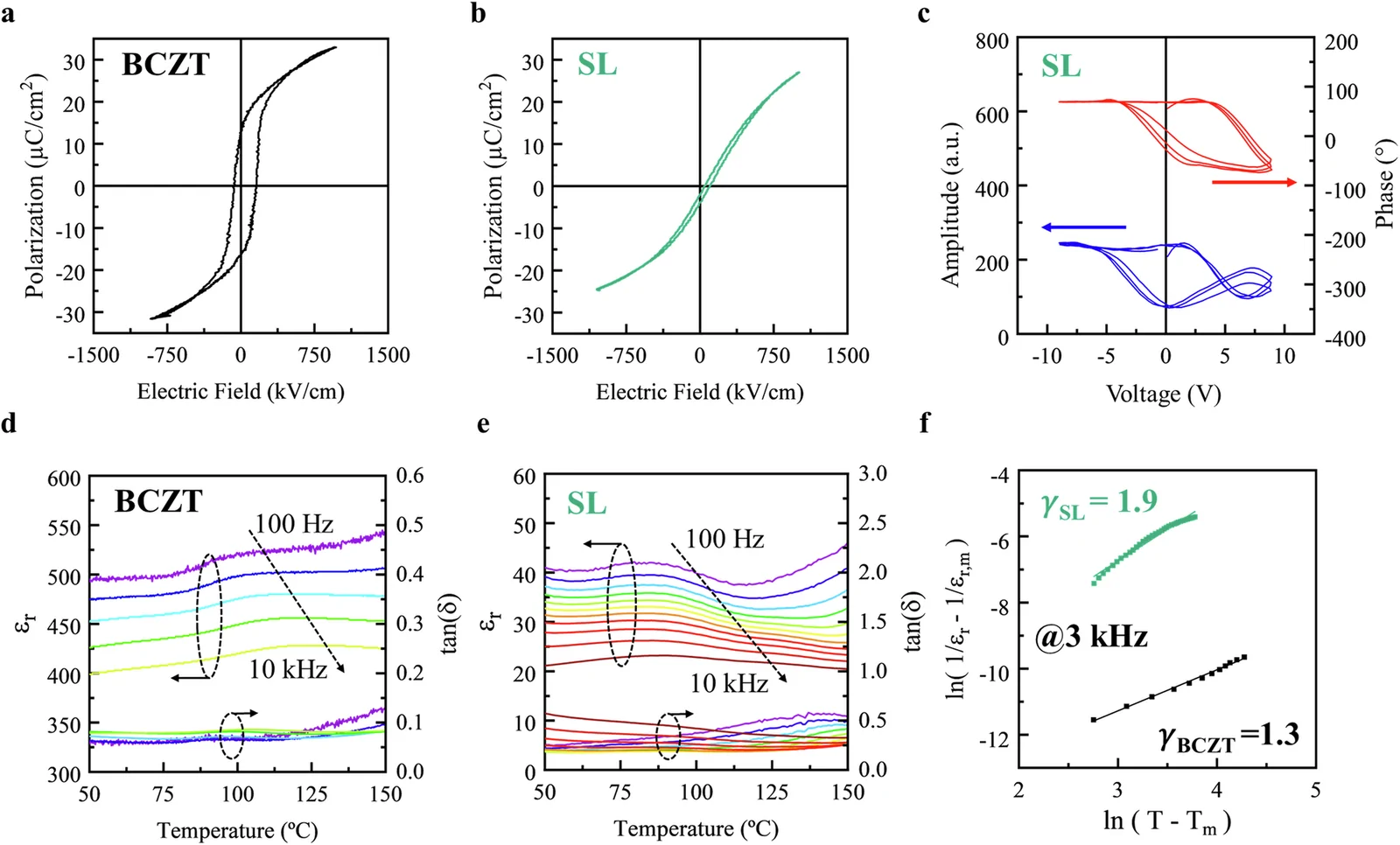
Ferroelectric and dielectric response of the BCZT film and BCZT/STO SL. **a** P-E hysteresis loop of the BCZT film capacitor. **b** P-E hysteresis loop of the SL measured under comparable electric field conditions. **c** Piezoresponse force microscopy (PFM) amplitude and phase hysteresis loops of the SL. **d** Temperature dependence of the dielectric permittivity (ε_r_) and loss tangent (tan δ) of the monolithic BCZT film measured at frequencies between 100 Hz and 10 kHz. **e** Temperature dependence of ε_r_ and tan δ of the SL under the same frequency range. **f** Modified Curie-Weiss analysis plotted at 3 kHz, yielding diffuseness parameters of γ_BCZT_ = 1.3 and γ_SL_ = 1.9.

The FE and dielectric properties of the BCZT and BCZT/STO SL were investigated. The P-E hysteresis loops of the BCZT film and the SL are shown in Fig. 3a, b, respectively. For the BCZT thin film, it is possible to observe a polarization-electric field (P-E) loop with an average maximum polarization (P_max,_ considering +P_max_ and -P_max_) and remnant polarization (P_r,_ considering +P_r_ and -P_r_) of 34 µC/cm^2^ and 14 µC/cm^2^, respectively. In addition, the average coercive field (E_c_) is around 160 kV/cm. In the case of the BCZT/STO SL, the P-E loops are ultra-slim. While the P_max_ is around 29 µC/cm^2^, the P_r_ and E_c_ are much lower, and their average values are close to 0 µC/cm^2^ and 110 kV/cm, respectively. We have also performed frequency-dependent P-E measurements from 0.5 to 2.5 kHz, under an applied electric field of 1100 kV/cm, for the BCZT/STO SL, as shown in Fig. S2a. The P-E loops remain slim and nearly overlapping throughout the investigated frequency range, confirming that the response is intrinsic.

In the case of the BCZT film and BCZT/STO SL capacitors an imprint effect is observed, which can be caused by the different work functions of the dissimilar top (Au) and bottom (LSMO) electrodes^32,33^, interface effect or substrate stress. PFM measurements were also performed to further confirm the imprint effect in the SL (Fig. 3c). The butterfly shape in the PFM amplitude as well as a hysteresis in the PFM phase demonstrate reversible switching under applied bias. Additionally, a strong imprint effect is observed, confirming the macroscopic P-E response. To understand the origin of the imprint effect, we have investigated the structural, strain and compositional properties of the STO/BCZT/STO/BCZT initial layers on top of the bottom LSMO electrode. Thus, four-dimensional (4D)-STEM experiments were conducted. The 4D-STEM data have been acquired on the HAADF-STEM image presented in Fig S3a. The corresponding 4D-STEM/automated crystal orientation mapping (ACOM) maps in Fig. S3b, c were obtained using a tetragonal BaTiO_3_-type perovskite structure as the crystallographic indexing model. Since the exact unstrained lattice parameters and room-temperature symmetry of the present BCZT composition are not independently known, these maps are interpreted primarily as relative spatial variations of the local lattice spacing rather than as an absolute quantitative determination of elastic strain. In addition, the values displayed within the STO layers are not quantitatively interpreted using the BCZT/BaTiO_3_-type reference. The most reproducible feature of the 4D-STEM analysis is the presence of an anisotropic and depth-dependent lattice distortion within the BCZT layers. The out-of-plane component exhibits a stronger variation, particularly in the lower BCZT layer close to the LSMO electrode, whereas the in-plane component is weaker and spatially heterogeneous. The magnitude of the lattice distortion varies over the first few nanometers from the BCZT/LSMO interface, indicating an interface-related structural gradient rather than a homogeneous biaxial strain state.

We note that the absolute magnitude of the mapped values may also be influenced by the choice of the reference lattice, local specimen thickness, residual preparation-induced surface damage, specimen bending or tilt, dynamical diffraction, and uncertainties in the fitting of the Bragg-disk positions. The cross-sectional specimen was prepared by conventional mechanical thinning and ion milling, and the different thinning rates of LSMO, BCZT, and STO may produce local thickness and surface-damage variations. Therefore, the maps are used to identify robust spatial trends in the lattice distortion, rather than to assign exact absolute strain values.

To further examine the mechanisms responsible for the relaxation of the interfacial distortion, high-resolution STEM images were analyzed at the BCZT/STO interfaces and within the BCZT layers. Burgers circuits constructed around representative lattice defects fail to close, confirming the presence of dislocations with non-zero Burgers vectors, as shown in Fig. S4. These defects can provide a mechanism for partial accommodation of the lattice mismatch and may contribute to the observed local relaxation. However, we recognize that differences in the boundary conditions of the successive BCZT layers, point-defect distributions, and local specimen-thickness effects may also influence the measured lattice parameters. Accordingly, dislocation formation is considered to be an important contribution to the relaxation process, rather than the exclusive origin. Furthermore, STEM-EDS elemental maps (Fig. 2 and S1) confirm the multilayer architecture and demonstrate clear elemental partitioning, with Ba, Ca and Zr concentrated within the BCZT layers, Sr and Ti associated with STO, and La and Mn localized within the LSMO electrode. Therefore, composition gradients^34^ can be excluded as the driving mechanism for the imprint effect, with the strain gradient^35^ instead of being the dominant contributor.

In order to further confirm if the strain gradient, 2-3 nm across the BCZT layer is responsible for the observed imprint effect, we have measured the P-E loops for a BCZT/STO SL with each individual layer being of thickness ~3 nm. Figure S5 shows the P-E loop and apart from the slim P-E loops typical of the RFE behavior, the imprint is no longer observed. Therefore, we conclude that a substrate-induced strain gradient arising from the lattice mismatch at the BCZT/LSMO interface is the primary factor responsible for the imprint effect, while the asymmetric electrode contribution is a negligible effect.

To further understand the remarkably different FE behavior of the BCZT and BCZT/STO SL, the dielectric properties of both samples were next investigated. Dielectric temperature-dependent measurements of the dielectric permittivity of the BCZT film and BCZT/STO SL are shown in Fig. 3d, e, respectively. The maximum value of dielectric permittivity decreases significantly in the BCZT/STO, which is consistent with the same behavior observed in BTO/STO SLs^28^. Also, the dielectric loss is higher in the BCZT/STO SL compared to BCZT thin films primarily due to the increased density of interfaces, which create strong depolarizing fields^36^. In addition, increasing numbers of interfaces may increase the number of defects that results in leakage that causes a greater loss. In addition, both samples show a broad peak in the temperature-dependent ε′ near 90 °C, associated with the tetragonal to cubic phase transition^37^. Moreover, the temperature of the maximum dielectric permittivity (T_m_) increases with increasing frequency of the applied field and there is a strong frequency dispersion of the dielectric permittivity around and below the temperature of maximum dielectric permittivity T_m_, indicating a RFE nature of the samples^38^.

To quantify the degree of diffuseness of both samples, the dielectric response was analyzed using the modified Curie-Weiss law^19^:

2 $$\frac{1}{\varepsilon }-\frac{1}{{\varepsilon }_{{{\rm{m}}}}}=\frac{{\left(T-{T}_{{{\rm{m}}}}\right)}^{\gamma }}{C}$$

where ε_m_ is the maximum dielectric permittivity and T_m_ is the corresponding temperature. The parameters γ and C are constants associated with the diffuseness of the phase transition, with γ = 1 for a normal FE and γ = 2 for an ideal RFE^19^. The resulting diffuseness parameter γ, extracted at 3 kHz, is presented in Fig. 3f. The BCZT film exhibits a value of γ = 1.3, indicating a weak RFE behavior, while the SL shows a significantly higher value of γ = 1.9, aproaching that of an ideal RFE. Therefore, the SL engineering strategy significantly enhances the RFE behavior in the BCZT. This enhancement could be explained by the presence of PNRs with varying sizes, randomly distributed in the BCZT film. This would lead to a broad distribution of relaxation times and a diffuse, frequency-dependent maximum in dielectric constant^38^. We examine this hypothesis in the next section.

### Visualizing polar nanoregions in BCZT/STO superlattices

To gain deeper insight into the atomic-scale structure of the BCZT layers in the SL and, consequently, of its RFE behavior, we analyzed the domain distribution by evaluating the projected displacements of the B-site cations (B = Ti/Zr) with respect to the lattice centers defined by their four nearest A-site (Ba/Ca) neighbors, using the high-resolution HAADF-STEM image shown in Fig. 1a. Owing to the Z^2^-contrast inherent to HAADF-STEM imaging, the atomic columns of the heavy A- and B-site cations appear as well-defined intensity maxima. We therefore implemented a Python-based routine in which each atomic column was fitted by a two-dimensional Gaussian function to determine the precise position of the intensity maximum associated with each B-site column. Within every unit cell, the displacement of the B-site cation was then calculated relative to the geometric center of the surrounding A-site cage, yielding a local polarization vector for each individual unit cell. Visualization and analysis of the displacement field were performed using the TopoTEM package^39^.

**Fig. 4:**
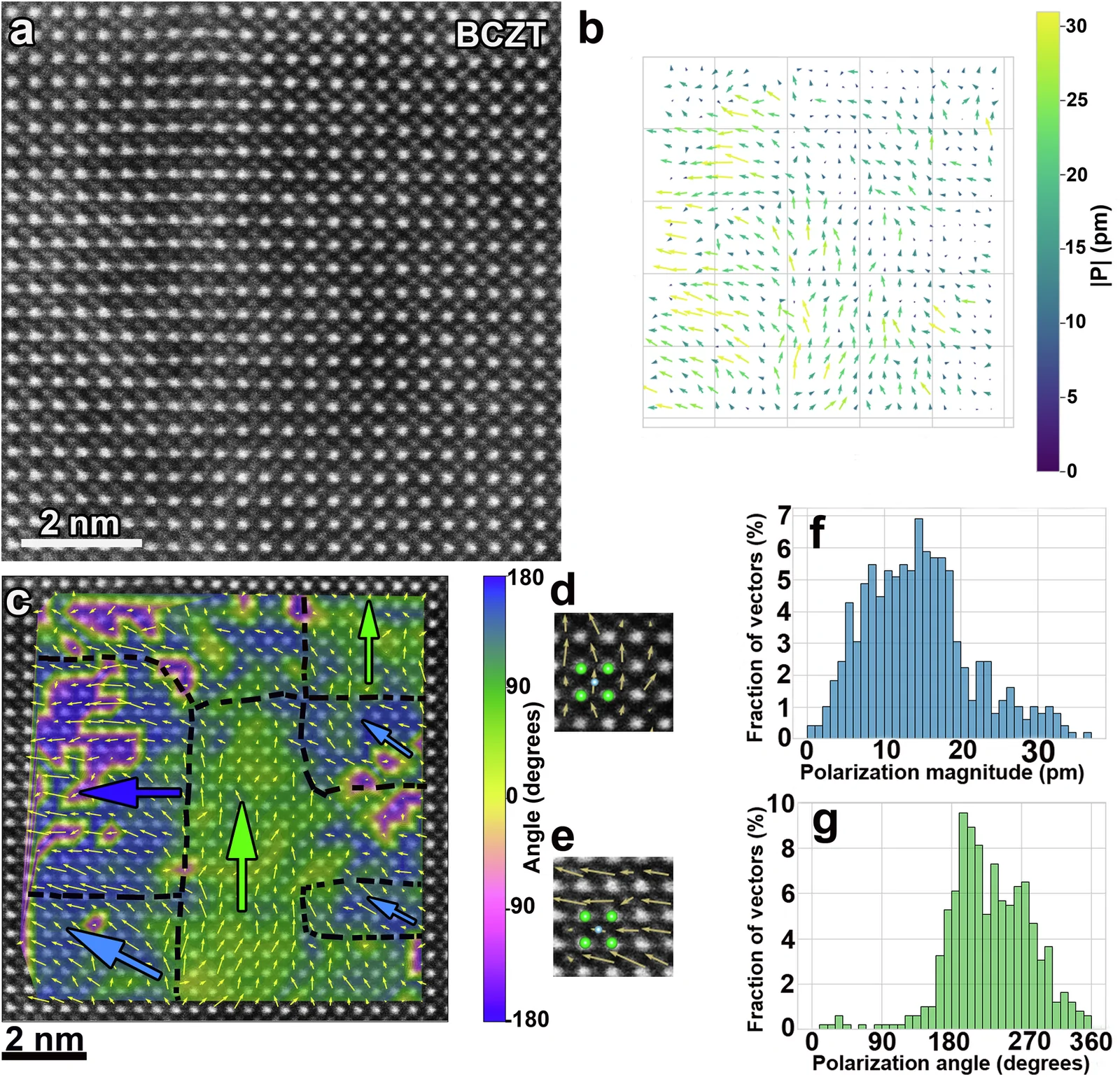
Atomic-scale polarization mapping and PNRs in the SL BCZT Layer. **a** Atomic resolution HAADF-STEM image of the BCZT layer in the SL. **b** Polarization vector map colored by magnitude. **c** Atomic-resolution HAADF-STEM polarization vector images along [100]c direction colored by their direction. Yellow arrows show the displacement of each B-site cation inside the unit cell. PNRs with the same B-site cation displacement vectors are circled as nanodomains using dark dashed lines. **d**, **e** Magnified images of selected areas from (**c**) with the BCZT unit cell overlapped. **f**, **g** Polarization vector magnitude and direction distribution of B-site cations obtained from the HAADF-STEM image (**c**).

The resulting polarization-vector map for the HAADF-STEM image in Fig. 4a is shown in Fig. 4b, where the vectors are color-coded by their magnitude. The corresponding histogram of polarization magnitudes, displayed in Fig. 4f, shows the distribution of the polarization vector magnitude of around 15−20 pm, which is a typical value for BaTiO_3_^40^ and BCZT-based ceramics^41^. This confirms that the observed displacements originate from genuine FE polarization rather than imaging artifacts or random thermal fluctuations.

To highlight the spatial organization of these displacements, the polarization-vector map, colored according to their orientation, is overlaid on the HAADF-STEM image in Fig. 4c. This representation clearly reveals the formation of PNRs, which are delineated by the dashed black contours. Within these regions, the polarization vectors are locally aligned, forming nanodomains with vertical (Fig. 4d), horizontal orientations (Fig. 4e), and areas showing inclined polarization directions.

The angular distribution of the polarization vectors further corroborates the presence of multiple polar variants. To account for the nematic symmetry of FE domains (p ≡ -p), the polarization angles were folded modulo 180° and then represented in the 0-360° range for visualization. The resulting angular distribution in Fig. 4g exhibits distinct peaks corresponding to different domain orientations, indicating the coexistence of multiple polarization variants with different characteristic length scales.

The presence of well-defined PNRs is of central importance for the ES performance of the BCZT layers. In RFE-like and weakly ordered FEs, PNRs facilitate large reversible polarization through their easy reorientation under an applied electric field, while simultaneously suppressing long-range FE order that would otherwise lead to large remanent polarization and high hysteretic losses^31^. To experimentally confirm that, we performed atomic displacement on atomic-resolution HAADF-STEM images acquired from a BCZT thin film. The cross-sectional STEM image in Fig. S6a shows the BCZT/LSMO/STO heterostructure, while the atomic-resolution HAADF-STEM image in Fig. S6b, acquired from the region indicated in Fig. S6a, resolves the BCZT lattice at the unit-cell level. The B-site displacement analysis was performed using the same procedure employed for the BCZT layers in the SL. As shown in Fig. S6c, the BCZT film exhibits spatially extended regions in which neighboring B-site displacement vectors retain a common orientation over considerably larger distances than those observed in the BCZT/STO SL. For clarity, vectors of uniform display length are used in this panel so that the spatial variation in orientation can be visualized independently of the displacement magnitude. The corresponding magnitude map is presented separately in Fig. S6d. Importantly, the displacement-magnitude distribution in the BCZT film is comparable to that obtained for the SL, with a median displacement of approximately 30 pm. Thus, the principal difference between the two systems is not the magnitude of the local polar displacement, but its spatial organization. Consistent with this observation, the angular distribution of the BCZT film is strongly concentrated around the dominant ~90° orientation, whereas a substantially broader distribution of local polar orientations is observed in the BCZT/STO SL. These observations demonstrate that nanoscale polar displacements are already present in the BCZT film and support a more spatially correlated polar state in the single-layer film, whereas the SL exhibits a more fragmented polar configuration. Our results indicate that SL engineering modifies the characteristic spatial correlation of the pre-existing PNRs in BCZT, consistent with confinement imposed by the periodic BCZT/STO interfaces.

The coexistence of smaller PNRs observed in BCZT/STO SL therefore provides valuable direct microscopic evidence for the smaller characteristic length of polar domains in SL compared with the single film that is highly favorable for high ŋ ES capacitors^11^.

### Energy storage properties and benchmarking

The ES characteristics of dielectric capacitors are evaluated from P-E hysteresis loops. The main parameters used to assess their performance include the ESD, η, and the applied electric field (E). These parameters can be calculated using the following equations:

3 $${W}_{{{\rm{stored}}}}=\int _{0}^{{P}_{{{\rm{m}}}}}E\,{{\rm{d}}}P$$

4 $${ESD}=\int _{{P}_{{{\rm{r}}}}}^{{P}_{{{\rm{m}}}}}E\,{{\rm{d}}}P$$

5 $$\eta=\frac{{ESD}}{{W}_{{{\rm{stored}}}}}$$

where W_stored_ represents the total stored energy density, P_m_ is the maximum polarization, and P_r_ denotes the remnant polarization^11^. Consequently, achieving high ES performance in dielectric capacitors requires a combination of high P_m_ and low P_r_ under a given applied electric field.

**Fig. 5:**
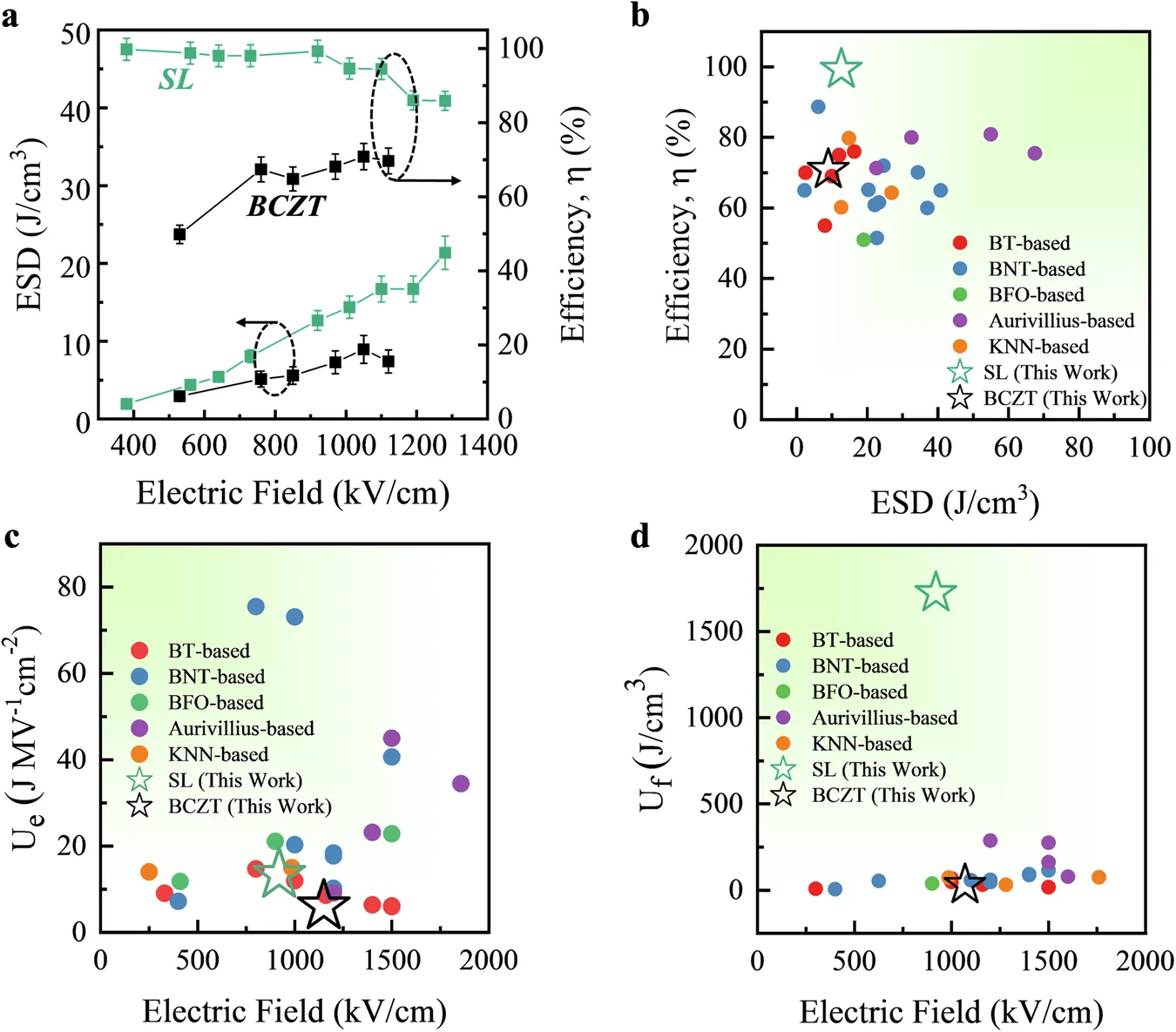
Electrostatic ES performance of the BCZT and BCZT/STO SL capacitors. **a** ESD and η as a function of electric field for the BCZT and BCZT/STO SL capacitors. Error bars are included taking into consideration that ten capacitors were measured for each sample. **b** η versus ESD for the BCZT and BCZT/STO SL compared with other lead-free dielectric systems. Comparison of the figure of merits **c** U_e_, and **d** U_f_ for the BCZT film and BCZT/STO SL with representative lead-free FE and RFE thin films reported in the literature. All data used in Fig. 5b–d is provided in Supporting Information (Table S1)^16,52–72^.

From the P-E hysteresis loops shown in Fig. 3a, b for the BCZT and the BCZT/STO SL capacitors, respectively, it is obvious that the SL approach can enhance the ESD and ŋ of the capacitor due to a higher difference between P_m_ and P_r_. This is due to enhanced RFE behavior arising from PNRs that can easily become randomly oriented in the absence of an applied electric field. The electric field dependence of the P-E loops for the BCZT and the BCZT/STO SL capacitors are shown in Fig. S7a, b, respectively. The ESD and ŋ values extracted from these P-E loops for the BCZT and the BCZT/STO SL samples, are plotted as a function of electric field in Fig. 5a. As the applied electric field increases, the ESD of the SL rises continuously, reaching values of 12.6 J/cm^3^ at 920 kV/cm with η over 99%. The high ESD and η values compared to ones achieved in the BCZT sample confirm the success of the SL strategy for achieving ultra-highly efficient ES capacitors. As observed in Fig. 5a, for higher applied fields the η value drops slightly, due to higher hysteresis losses^42^. At the highest applied field of 1280 kV/cm, the ESD reaches 20.4 J/cm^3^, with an η value of 86%. Therefore, the ESD and η values in the BCZT/STO SL are about 40% higher than the values obtained in the BCZT film. As already noted, this can be ascribed to the enhanced RFE behavior. In addition, the BCZT/STO SL capacitor demonstrates a stable ES performance, up to 2 × 10^8^ cycles, as shown in Fig. S2b. Furthermore, as shown in Fig. 5a, a higher breakdown electric field is obtained in the BCZT/STO SL compared to the BCZT film. This can be explained by the presence of multiple interfaces and epitaxial strain which produce physical barriers and energy barriers, respectively^43^, in addition to a better distribution of electrical and mechanical stresses which significantly delays the dielectric breakdown^44^.

Figure 5b shows the ESD and η values for the BCZT film and the BCZT/STO SL, compared to different lead-free FE and RFE thin films in the literature (for applied electric fields in the range 250-1750 kV/cm). Table S1 shows details of electric field and corresponding ESD and η values of different capacitors based on representative lead-free FE and RFE thin films reported in the literature. It is observed from Fig. 5b that the ESD and η values for the BCZT films are similar to many other systems in the literature. On the other hand, for the BCZT/STO SL films, η is more than 10% higher than the highest value in the literature, which is for 0.5(Bi_0.45_Na_0.45_Ba_0.1_TiO_3_) (BNT)-0.5Bi(Mg_1/3_Nb_2/3_)O_3_-based capacitors and at least 20% higher than for other FE and RFE thin film capacitors. Thus, for the SL film, less energy is lost as heat, which is crucial for pulsed power applications.

The charge-discharge response was also evaluated from transient current-time (I-t) measurements performed using square voltage pulses. The discharged energy density was calculated from the time-integrated Joule energy dissipated through the discharge resistance, using:

6 $${W}_{{{\rm{dis}}}}\left(t\right)=\frac{R}{V} \int _{0}^{t}I{\left(t\right)}^{2}\,{{\rm{d}}}t$$

where R is the total discharge resistance (R = 100 Ω), I(t) is the measured time-dependent discharge current, and V is the active volume of the capacitor. The resulting W_dis_ (t) curves, shown in Fig. S2(c), exhibit a rapid increase followed by a well-defined saturation plateau. The plateau value corresponds to the discharged energy density at each applied field. As expected, the W_dis_ plateau increases with increasing nominal electric field, confirming that more energy is released from the capacitor when it is charged to higher fields. At the highest applied field of 1100 kV/cm, we obtain a W_dis_ value of 16.3 J/cm^3^, which is close to the ESD value of 16.7 J/cm^3^ obtained from the P-E loop. The minimal difference is due to contact resistance and internal serial resistances within the film, including grain boundaries and interfaces, which can dissipate part of the stored energy^45^.

A Weibull statistical analysis of the breakdown field for the BCZT/STO SL is shown in Fig. S2d. Breakdown measurements were performed on n = 10 BCZT/STO SL capacitors and a breakdown field value of E_BD_ = 1342 ± 12 kV/cm and a Weibull modulus of β = 18.8 ± 2.9 were achieved. The uncertainty in E_BD_ was calculated by propagating the uncertainty of the linear fit parameters, including the covariance between the intercept and slope. The relatively high Weibull modulus indicates a narrow breakdown distribution and excellent capacitor reproducibility for the BCZT/STO SL^46,47^.

Figure S2e, f illustrates the temperature-dependent unipolar P-E loops and the ES performance for the BCZT/STO SL. The response is stable in the measured temperature range. However, it is possible to observe that the loops become linear after 85 °C, which is close to Tm, the temperature of maximum permittivity observed in the dielectric measurements. Nevertheless, the BCZT/STO SL shows stable ES performance up to 110 °C, with no obvious performance degradation.

We have estimated the ESD normalized by the applied electric field as follows:

7 $${U}_{e}=\frac{{ESD}}{E}$$

As shown in Fig. 5c, the BCZT/STO SL exhibits a competitive performance within the low-to-moderate field range considered. Although the SL does not show the highest U_e_ among all reported lead-free thin film capacitors, it combines a competitive U_e_ with a significantly higher efficiency than most comparable lead-free FE and RFE systems.

Another important parameter is how effectively the ESD can be released (U_f_)^48^. This is defined as follows:

8 $${U}_{f}\,\left({\mbox{J}}/{\mbox{c}}{{\mbox{m}}}^{3}\right)=\frac{{ESD}}{1-\eta }$$

Figure 5d benchmarks the U_f_ values for BCZT and BCZT/STO SL capacitors compared to representative lead-free FE and RFE thin films reported in the literature (Table S1 shows further details of the comparison). The BCZT/STO SL has a 5x higher U_f_ value under comparable electric fields, placing it as the highest-performing lead-free oxide system. We have considered the errors in ESD and η to be 1.97 J/cm^3^ and 2.6%, respectively, considering the evaluation of the energy storage performance recorded for 20 different measurements. The uncertainty in U_f_ was estimated and the U_f_ is equal to 1723±273 J/cm^3^, which means that the error in U_f_ is around 16%. This calculation confirms that the central value of U_f_ reported in the table is correctly calculated from the measured ESD and the corresponding efficiency. Overall, the reported U_f_ is by far the highest value reported in the literature for representative lead-free FE and RFE thin films. Overall, the BCZT/STO SL shows a much better ES performance than the BCZT film, and many reported lead-free FE and RFE thin film capacitors. Thus, these results suggest that SL engineering provides an effective strategy for tuning PNR size and, consequently, the relaxor response of BCZT, which is harnessed in our work to obtain improved ES properties.

In this work, we have demonstrated that superlattice engineering of an epitaxial 0.5Ba(Zr_0.2_Ti_0.8_)O_3_-0.5(Ba_0.7_Ca_0.3_)TiO_3_/SrTiO_3_ system provides an effective strategy to tailor and strongly improve relaxor ferroelectric performance. Thus, much enhanced relaxor behavior was observed and was shown to arise from the formation of polar nanoregions which arise from the superlattice configuration. The nanoscale polarization configuration leads to slim hysteresis loops, and highly reversible polarization. The enhanced ES performance is also linked to an imprint effect caused by substrate-induced strain gradient imposed by the bottom electrode that caused a built-in field. The combination of the polar nanoregions and the imprint effect enables an optimized balance of energy storage efficiency and recoverable energy release at low applied electric fields/voltages. Our thin film system performance far exceeds single-layer BCZT films and other wide-ranging lead-free ferroelectric and relaxor ferroelectric thin films. Overall, our SL engineering approach holds great promise for realizing very high-performance lead-free dielectric ES capacitors for pulsed power electronics.

## Methods

### Thin film growth and device fabrication

Epitaxial thin films of BCZT and a BCZT/STO SL, both with a total thickness of 200 nm, were deposited on single-crystal SrTiO_3_ (001) substrates buffered with a conductive La_0.7_Sr_0.3_MnO_3_ (LSMO) bottom electrode using pulsed laser deposition (PLD). For the case of the BCZT/STO SL, each individual layer has a thickness of ~15 nm. The thin films were deposited by PLD using a KrF excimer laser (λ = 248 nm). The laser beam was focused onto ceramic targets and operated at a repetition rate of 2 Hz with a fluence of ~1.5 J/cm^2^. The depositions were carried out at a substrate temperature of 750 °C under an oxygen pressure of 5×10^−2^ mbar. After deposition, the samples were annealed in situ at 775 °C for 20 min under an oxygen flux of 0.1 mbar to improve crystallinity and reduce oxygen vacancy concentration. Subsequently, the films were cooled to room temperature at a rate of 10 °C/min while maintaining the same oxygen flux.

### Structural characterization

The structural properties of the samples were investigated by X-ray diffraction (XRD) using a Bruker D8 Discover diffractometer with Cu Kα radiation (λ = 1.54056 Å). Transmission electron microscopy (TEM) characterization was performed on cross-sectional samples prepared by mechanical cutting and polishing, followed by ion milling using a precision ion polishing system (PIPS). High-angle annular dark-field scanning transmission electron microscopy (HAADF-STEM) imaging and energy-dispersive X-ray spectroscopy (EDS) mapping were carried out using a probe-corrected Thermo Fisher Spectra 300 transmission electron microscope operated at an acceleration voltage of 200 kV and equipped with a Super-X EDS detector. A convergence semi-angle of 15.7 mrad was used, providing a depth of focus of approximately 10 nm. The HAADF detector collection angles ranged from 72 to 200 mrad using a camera length of 91 mm. These conditions enabled atomic-resolution imaging and high-sensitivity elemental mapping across the SL interfaces. Atomic-resolution HAADF-STEM images were further analyzed to determine the displacement of B-site cations relative to the surrounding A-site lattice. The atomic column positions were obtained by fitting the intensity maxima using a two-dimensional Gaussian function, allowing calculation of local polarization vectors for each unit cell. The analysis was performed using the TopoTEM package.

### Second-harmonic generation measurements

Second-harmonic generation (SHG) measurements were performed using a polarization-resolved SHG microscope in epi-reflection geometry. The experimental setup for the polarized second harmonic generation is given in Fig. S8. Excitation was provided by a mode-locked Ti:sapphire laser (Mira 900, Coherent, Saxonburg, PA, USA) operating at 76 MHz. Incident pulses had a central wavelength tuned to 794 nm, while the pulse duration and incident pulse energy were approximately 120 fs and 66 pJ at the sample. The beam was focused with a 40× Olympus Plan Achromat objective (NA = 0.65, f = 4.5 mm), providing a diffraction-limited estimated focal spot diameter of ~1.3 µm and an incident fluence of ~10 mJ/cm^2^.

The incident polarization was set using calcite polarizers and a zero-order half-wave plate (800 nm). The SHG polarization was analyzed with an equivalent polarizer/analyzer set and a zero-order half-wave plate for 400 nm. Residual fundamental light was suppressed using a short-pass dichroic beamsplitter (FP720-SDi01, Semrock, Rochester, NY, USA) together with a 40 nm wide band-pass filter centered at 400 nm (FBH400-40, Thorlabs, Newton, NJ, USA). The SHG signal was dispersed by a 300 mm imaging spectrometer (Shamrock 303i, Andor, Oxford, UK) equipped with a 2400 lines/mm grating and detected on a cooled CCD camera (Newton 920, Andor, Oxford, UK). The incident and analyzed SHG polarizations were scanned in 10° steps by rotating the excitation and analyzer half-wave plates in 5° increments.

### Dielectric measurements

For the electrical measurements, circular Au top electrodes (diameter 0.25 mm) were deposited on the film surface by thermal evaporation using a shadow mask to define the capacitor structures with controlled geometry. Temperature-dependent dielectric measurements were carried out using a precision LCR meter (Agilent E4980A) over the frequency range from 100 Hz to 10 kHz. The measurements were performed from room temperature up to 150 °C with a heating rate of 0.5 °C min^-1^. Temperature control was achieved using a PL 706 PID controller (Polymer Laboratories, Thermal Sciences, Epsom, UK) coupled to a programmable furnace. The sample temperature was monitored using a PT100 resistance thermometer (Omega, Manchester, UK). An AC excitation voltage of 50 mV was applied during the measurements. Shielded electrical connections were used to minimize parasitic impedance contributions from the measurement setup. The complex permittivity, defined as ε = ε′−iε″, where ε′ and ε″ are the real and imaginary parts, respectively, was then determined from the measured capacitance C and loss tangent tan δ using the parallel-plate capacitor relation and dielectric loss definition^49^.

### Ferroelectric and piezoelectric measurements

Polarization-electric field (P-E) hysteresis loops were measured at room temperature using a modified Sawyer-Tower circuit, enabling evaluation of the FE response and ES properties. The charge-discharge performance was measured using square pulses, and the corresponding current was measured across a 100 Ω resistor connected in series with the sample.

Piezoresponse force microscopy (PFM) experiments were carried out using an MFP-3D system (Oxford Instruments). Pt-coated n-type silicon cantilevers (BudgetSensors, model Multi75E-G) were employed for the measurements. To improve detection sensitivity, the dual AC resonance tracking (DART)^50^ technique was applied together with a compensation bias voltage^51^.

## Supplementary information

Supplementary Information

Transparent Peer Review file

## Data Availability

All raw data generated during the current study are available from the corresponding author upon request.

## References

[1] Pan, H. et al. Ultrahigh energy storage in superparaelectric relaxor ferroelectrics. *Science* **374**, 100–104 (2021). 10.1126/science.abi7687 34591628

[2] Aramberri, H., Fedorova, N. S. & Íñiguez, J. Ferroelectric/paraelectric superlattices for energy storage. *Sci. Adv.* **8**, eabn4880 (2022). 10.1126/sciadv.abn4880 PMC9348786 35921413

[3] Junquera, J. et al. Topological phases in polar oxide nanostructures. *Rev. Mod. Phys.* **95**, 025001 (2023).

[4] Zubko, P., Gariglio, S., Gabay, M., Ghosez, P. & Triscone, J.-M. Interface physics in complex oxide heterostructures. *Annu. Rev. Condens. Matter Phys.* **2**, 141–165 (2011).

[5] Yadav, A. K. et al. Observation of polar vortices in oxide superlattices. *Nature* **530**, 198–201 (2016). 10.1038/nature16463 26814971

[6] Kang, R. et al. Ultra-high energy storage in relaxor ferroelectric MLCCs at elevated temperatures via entropy modulated strain heterogeneity. *Nat. Commun.* **16**, 9065 (2025). 10.1038/s41467-025-64113-7 PMC12518529 41083435

[7] Yin, C. et al. Mimicking antiferroelectrics with ferroelectric superlattices. *Adv. Mater.* **36**, 2403985 (2024). 10.1002/adma.202403985 39318084

[8] Liu, Y. et al. Phase-field simulations of tunable polar topologies in lead-free ferroelectric/paraelectric multilayers with ultrahigh energy-storage performance. *Adv. Mater.* **34**, 2108772 (2022). 10.1002/adma.202108772 35034410

[9] Dan, T. et al. Strain-engineered phase diagrams in (SrTiO3)8/(BaTiO3)8 superlattices: toward néel skyrmions and energy storage. *Nanomaterials* **16**, 582 (2026). 10.3390/nano16100582 PMC13209491 42188477

[10] Ortega, N. et al. Relaxor-ferroelectric superlattices: high energy density capacitors. *J. Phys. Condens. Matter* **24**, 445901 (2012). 10.1088/0953-8984/24/44/445901 23053172

[11] Nguyen, M. D. et al. Toward design rules for multilayer ferroelectric energy storage capacitors - a study based on lead-free and relaxor-ferroelectric/paraelectric multilayer devices. *Adv. Mater.* **36**, 2402070 (2024). 10.1002/adma.202402070 38616493

[12] Zhang, X. et al. Ultra-thin multilayer films for enhanced energy storage performance. *Nano Energy* **121**, 109271 (2024).

[13] Liu, Y. et al. Ultrahigh capacitive energy storage through dendritic nanopolar design. *Science* **388**, 211–216 (2025). 10.1126/science.adt2703 40208979

[14] Jayakrishnan, A. R. et al. Are lead-free relaxor ferroelectric materials the most promising candidates for energy storage capacitors? *Prog. Mater. Sci.* **132**, 101046 (2023).

[15] Hanani, Z. et al. Unlocking high capacitive energy-density in Sm-doped Pb(Mg1/3Nb2/3)O3-PbTiO3 thin films via strain and domain engineering. *J. Mater. Chem. C* **13**, 7140–7149 (2025). 10.1039/d5tc00384a PMC11870079 40026676

[16] Konsago, S. W. et al. Engineering the microstructure and functional properties of 0.5Ba(Zr0.2Ti0.8)O3-0.5(Ba0.7Ca0.3)TiO3 thin films. *ACS Appl. Electron. Mater.* **6**, 4467–4477 (2024).

[17] Karmegam, S. P. & Murugavel, P. Lead-free BaTiO3-based relaxor ferroelectric thin film rendering rapid discharge rate for pulsed power energy application. *APL Energy* **2**, 016108 (2024).

[18] Nguyen, M. D., Houwman, E. P., Do, M. T. & Rijnders, G. Relaxor-ferroelectric thin film heterostructure with large imprint for high energy-storage performance at low operating voltage. *Energy Storage Mater* **25**, 193–201 (2020).

[19] Ribeiro, D. J. M. et al. Engineering Pb-free relaxor ferroelectric thin films for low voltage energy storage applications. *Nanoscale* **17**, 19794–19805 (2025). 10.1039/d5nr02537k 40813330

[20] Yu, H. et al. Design of polymorphic heterogeneous shell in relaxor antiferroelectrics for ultrahigh capacitive energy storage. *Nat. Commun.* **16**, 886 (2025). 10.1038/s41467-025-56316-9 PMC11751392 39837866

[21] Yang, Y. et al. Giant energy storage density with ultrahigh efficiency in multilayer ceramic capacitors via interlaminar strain engineering. *Nat. Commun.* **16**, 1300 (2025). 10.1038/s41467-025-56605-3 PMC11790901 39900915

[22] Chatterjee, S. et al. Interfacial strain induced giant magnetoresistance and magnetodielectric effects in multiferroic BCZT/LSMO thin film heterostructures. *J. Appl. Phys.* **135**, 184101 (2024).

[23] Tang, X.-G. & Chan, H. L.-W. Effect of grain size on the electrical properties of (Ba,Ca)(Zr,Ti)O3 relaxor ferroelectric ceramics. *J. Appl. Phys.* **97**, 034109 (2005).

[24] Wang, C. et al. Ultrahigh breakdown strength and exceptional energy storage performance in lead-free BCZT-based relaxor ferroelectric ceramics via composition-process synergistic optimization. *J. Alloys Compd.* **1064**, 187991 (2026).

[25] Chatterjee, S. et al. Role of oxygen vacancies on the low-temperature dielectric relaxor behavior in epitaxial Ba0.85Ca0.15Ti0.9Zr0.1O3 thin films. *Phys. Rev. Mater.* **5**, 064415 (2021).

[26] Nadaud, K. et al. History-dependent and frequency-dependent dielectric nonlinearities induced by polar nanoregions in a thin film of 0.5(Ba0.7Ca0.3TiO3)-0.5(BaZr0.2Ti0.8O3). *Phys. Rev. B* **112**, 014202 (2025).

[27] Khestanova, E. et al. Untangling electrostatic and strain effects on the polarization of ferroelectric superlattices. *Adv. Funct. Mater.* **26**, 6446–6453 (2016).

[28] Lupi, E. et al. Engineering relaxor behavior in (BaTiO3)n/(SrTiO3)n Superlattices. *Adv. Mater.* **35**, 2302012 (2023). 10.1002/adma.202302012 37433562

[29] Razumnaya, A. G. et al. Phase transition in ferroelectric BaTiO3/SrTiO3 superlattice: Raman spectroscopy studies. *Ferroelectrics* **501**, 61–69 (2016).

[30] Sarott, M. F., Rossell, M. D., Fiebig, M. & Trassin, M. Multilevel polarization switching in ferroelectric thin films. *Nat. Commun.* **13**, 3159 (2022). 10.1038/s41467-022-30823-5 PMC9174202 35672404

[31] Cherifi-Hertel, S. et al. Shedding light on non-Ising polar domain walls: Insight from second harmonic generation microscopy and polarimetry analysis. *J. Appl. Phys.* **129**, 081101 (2021).

[32] Chen, Y.-W. et al. An electrode design strategy to minimize ferroelectric impRINT EFFECT. *Adv. Sci*. **12**, e70011 (2025). 10.1002/advs.70011 PMC12362776 40402000

[33] Liu, F., Fina, I., Bertacco, R. & Fontcuberta, J. Unravelling and controlling hidden imprint fields in ferroelectric capacitors. *Sci. Rep.* **6**, 25028 (2016). 10.1038/srep25028 PMC4848548 27122309

[34] Afanasjev, V. P. et al. Polarization and self-polarization in thin PbZr1−xTixO3 (PZT) films. *J. Phys. Condens. Matter* **13**, 8755–8763 (2001).

[35] Jeon, B. C. et al. Flexoelectric effect in the reversal of self-polarization and associated changes in the electronic functional properties of BiFeO3 thin films. *Adv. Mater.* **25**, 5643–5649 (2013). 10.1002/adma.201301601 23897638

[36] Zubko, P. et al. Electrostatic coupling and local structural distortions at interfaces in ferroelectric/paraelectric superlattices. *Nano Lett* **12**, 2846–2851 (2012). 10.1021/nl3003717 22591200

[37] Liu, W. & Ren, X. Large piezoelectric effect in pb-free ceramics. *Phys. Rev. Lett.* **103**, 257602 (2009). 10.1103/PhysRevLett.103.257602 20366285

[38] Li, F., Zhang, S., Damjanovic, D., Chen, L.-Q. & Shrout, T. R. Local structural heterogeneity and electromechanical responses of ferroelectrics: learning from relaxor ferroelectrics. *Adv. Funct. Mater.* **28**, 1801504 (2018).

[39] O’Connell, E. N. et al. TopoTEM: a python package for quantifying and visualizing scanning transmission electron microscopy data of polar topologies. *Microsc. Microanal.* **28**, 1444–1452 (2022). 10.1017/S1431927622000435 35318910

[40] Cavanagh, A. E. et al. Effect of Stoichiometry on the Structure and Polarization of BaTiO3. *Adv. Opt. Mater.* **13**, 2501037 (2025).

[41] Xie, A. et al. Superior capacitive energy storage of batio3-based polymorphic relaxor ferroelectrics engineered by mesoscopically chemical homogeneity. *Adv. Sci.* **12**, 2502916 (2025). 10.1002/advs.202502916 PMC12279175 40411412

[42] Zou, X. et al. Energy storage performance of BNT-based relaxor ferroelectric thin films by introducing highly ergodic components. *Ceram. Int.* **52**, 34422–34431 (2026).

[43] Sun, Z. et al. Interface engineering in ferroelectrics: From films to bulks. *J. Alloys Compd.* **909**, 164735 (2022).

[44] Wu, Z., Liu, Y., Zhou, J., Zhao, H. & Qin, Z. Interface structure, dielectric behavior and temperature stability of Ba(Mg1/3Ta2/3)O3/PbZr0.52Ti0.48O3 thin films. *Materials* **16**, 6358 (2023). 10.3390/ma16196358 PMC10573921 37834494

[45] Kobald, H., Kobald, A. M., Gindel, T., Panzic, I. & Deluca, M. Lead-free relaxor ferroelectric thin films with enhanced energy density through process optimization. *Commun. Mater.* **6**, 250 (2025).

[46] Kobald, A. M., Kobald, H., Gindel, T., Panzic, I. & Deluca, M. Ultrahigh energy storage in process-engineered NaNbO₃-based thin films with superior thermal and cyclic stability. *Sci. Rep.* **15**, 21617 (2025). 10.1038/s41598-025-05243-2 PMC12216296 40594500

[47] Pan, H. et al. Giant energy density and high efficiency achieved in bismuth ferrite-based film capacitors via domain engineering. *Nat. Commun.* **9**, 1813 (2018). 10.1038/s41467-018-04189-6 PMC5940880 29739938

[48] Zhao, W. et al. Advanced stability and energy storage capacity in hierarchically engineered Bi0.5Na0.5TiO3-based multilayer capacitors. *Nat. Commun.* **16**, 6549 (2025). 10.1038/s41467-025-61936-2 PMC12267464 40670352

[49] Rodriguez, B. J., Callahan, C., Kalinin, S. V. & Proksch, R. Dual-frequency resonance-tracking atomic force microscopy. *Nanotechnology* **18**, 475504 (2007).

[50] Jesse, S., Baddorf, A. P. & Kalinin, S. V. Switching spectroscopy piezoresponse force microscopy of ferroelectric materials. *Appl. Phys. Lett.* **88**, 062908 (2006). 10.1103/PhysRevLett.96.237602 16803404

[51] Tan, H. et al. A transversal approach to predict surface charge compensation in piezoelectric force microscopy. *Appl. Surf. Sci.* **607**, 154991 (2023).

[52] Lakouader, A. et al. BCZT/LSMO/BCZT sandwich film: Toward high-temperature energy storage capacitors. *Materialia* **38**, 102309 (2024).

[53] Lakouader, A. et al. Thermally stable electrostatic energy storage in X9R-Type BaTiO3-based thin films elaborated by chemical solution deposition. *J. Energy Storage* **141**, 119421 (2026).

[54] Angermann, M. et al. High energy density and reliable BaZrxTi1−xO3 thin film capacitors from novel aqueous chemical solution deposition. *J. Eur. Ceram. Soc.* **45**, 117465 (2025).

[55] Luo, X. et al. Flexible lead-free Na0.5Bi0.5TiO3-based film capacitor with stable energy storage performances. *J. Mater. Sci. Mater. Electron.* **34**, 604 (2023).

[56] Wang, F., Zhu, C. & Zhao, S. Good energy storage properties of Na0.5Bi0.5TiO3 thin films. *J. Alloys Compd.* **869**, 159366 (2021).

[57] Wu, S. et al. Enhanced piezoelectricity and energy storage performances of Fe-doped BNT-BKT-ST thin films. *Ceram. Int.* **44**, 21289–21294 (2018).

[58] Chen, P. et al. Enhanced dielectric and energy-storage properties in BiFeO3-modified Bi0.5(Na0.8K0.2)0.5TiO3 thin films. *Ceram. Int.* **43**, 13371–13376 (2017).

[59] Wu, J. et al. Excellent energy storage and photovoltaic performances in bi0.45na0.45ba0.1tio3-based lead-free ferroelectricity thin film. *Ceramics* **7**, 1043–1052 (2024).

[60] Quan, N. D., Toan, T. Q. & Hung, V. N. Influence of crystallization time on energy-storage density and efficiency of lead-free Bi0.5(Na0.8K0.2)0.5TiO3 thin films. *Adv. Condens. Matter Phys.* **2019**, 4949365 (2019).

[61] Wu, Y., Hu, Y., Wang, X., Zhong, C. & Li, L. Temperature- and frequency-dependent dielectric response and energy-storage performance in high (100)-oriented Sc-doped (Na0.85K0.15)0.5Bi0.5TiO3 films. *RSC Adv* **7**, 51485–51494 (2017).

[62] Sun, M. et al. Enhanced energy storage performance in BNKT-based lead-free thin films at low electric fields. *ACS Appl. Electron. Mater.* **7**, 11284–11293 (2025).

[63] Liu, X., Yao, Y., Wang, X., Zhao, L. & San, X. Energy storage performance of (na0.5bi0.5)tio3 relaxor ferroelectric film. *Coatings* **14**, 801 (2024).

[64] Sun, N., Li, Y., Liu, X. & Hao, X. High energy-storage density under low electric field in lead-free relaxor ferroelectric film based on synergistic effect of multiple polar structures. *J. Power Sources* **448**, 227457 (2020).

[65] Hu, Y. et al. High energy storage performance in lead-free BiFeO3-BaTiO3 ferroelectric thin film fabricated by pulsed laser deposition. *AIP Adv* **9**, 085005 (2019).

[66] Keum, Y. H., Shin, H. W. & Son, J. Y. Improvement of energy storage performance by controlling the crystallinity of Aurivillius BaBi4Ti4O15 thin films. *J. Korean Ceram. Soc.* **60**, 959–966 (2023).

[67] Song, D. & Yang, J. Lead-free Ba2Bi4Ti5O18 thin film capacitors for energy storage applications. *Results Phys* **13**, 102175 (2019).

[68] Liu, Q. et al. Intercalation strategy induced superior energy storage performance in Aurivillius Bi6Ti3FeAlO18 film under low and medium electric fields. *J. Materiomics* **12**, 101113 (2026).

[69] Zhou, J. et al. Optimized energy storage performance of SBT-based lead-free relaxor ferroelectric thin film. *J. Mater. Sci. Mater. Electron.* **34**, 1294 (2023).

[70] Sun, Y., Zhou, Y., Lu, Q. & Zhao, S. High energy storage efficiency with fatigue resistance and thermal stability in lead-free Na0.5K0.5NbO3/BiMnO3 solid-solution films. *Phys. Status Solidi RRL* **12**, 1700364 (2018).

[71] Cheng, Y.-Y. -S. et al. All-inorganic flexible (K, Na)NbO3-BASED LEAD-FREE PIEZOELECTRIC THIN FILMS SPIN-COATED ON METALLIC FOIls. *ACS Appl. Mater. Interfaces* **13**, 39633–39640 (2021). 10.1021/acsami.1c11418 34382760

[72] Zhao, C., Meng, X., Wang, W. & Zhou, Y. Energy storage performance of (K, Na)NbO3 ferroelectric thin films with Mn-Ta and Mn-Ti co-doping. *Ceram. Int.* **45**, 13772–13779 (2019).

