# Supplementary material for: Superlattice Engineering in Relaxor Ferroelectric-based Capacitors for Ultra-Highly Efficient Recoverable Energy Storage Density
Source: Nat Commun. 2026 Oct 5;17:11338. doi: 10.1038/s41467-026-77971-6 (PMC13728193; doi:10.1038/s41467-026-77971-6)
Supplement: Supplementary file 1 [file 41467_2026_77971_MOESM1_ESM.pdf]

Supporting Information

Superlattice Engineering in Relaxor Ferroelectric-based Capacitors for Ultra-Highly Efficient Recoverable Energy Storage Density

Matilde M. G. S. Oliveira[1,2], Ampattu R. Jayakrishnan[1,2], Duarte J. M. Ribeiro[1,2], Tiago Rodrigues[1,2], Jingye Zou,[3] Surya K. P. Nair[1,2], Raluca Negrea[4,5], Marian C. Istrate[5], Manuel J. L. F. Rodrigues[1,2], Michael Belsley[1,2], Bernardo G. Almeida[1,2], Mario Pereira[1,2], Judith L. MacManus-Driscoll[6], Ignasi Fina[3], Florencio Sánchez[3], Luís Marques[1,2], José P. B. Silva[1,2,*]

[1]Physics Center of Minho and Porto Universities (CF-UM-UP), University of Minho, Campus de Gualtar, 4710-057 Braga, Portugal

[2]Laboratory of Physics for Materials and Emergent Technologies, LapMET, University of Minho, 4710-057 Braga, Portugal

[3]Institut de Ciència de Materials de Barcelona (ICMAB-CSIC), Campus UAB, Bellaterra 08193, Barcelona, Spain

[4]Brunel Centre for Advanced Solidification Technology (BCAST), Brunel University London, Uxbridge, Middlesex UB8 3PH, UK

[5]National Institute of Materials Physics, 405A Atomistilor, 077125 Magurele, Romania

[6]Department of Materials Science & Metallurgy, University of Cambridge, 27 Charles Babbage Road, Cambridge CB3 0FS, United Kingdom

* Corresponding author e-mail: josesilva@fisica.uminho.pt

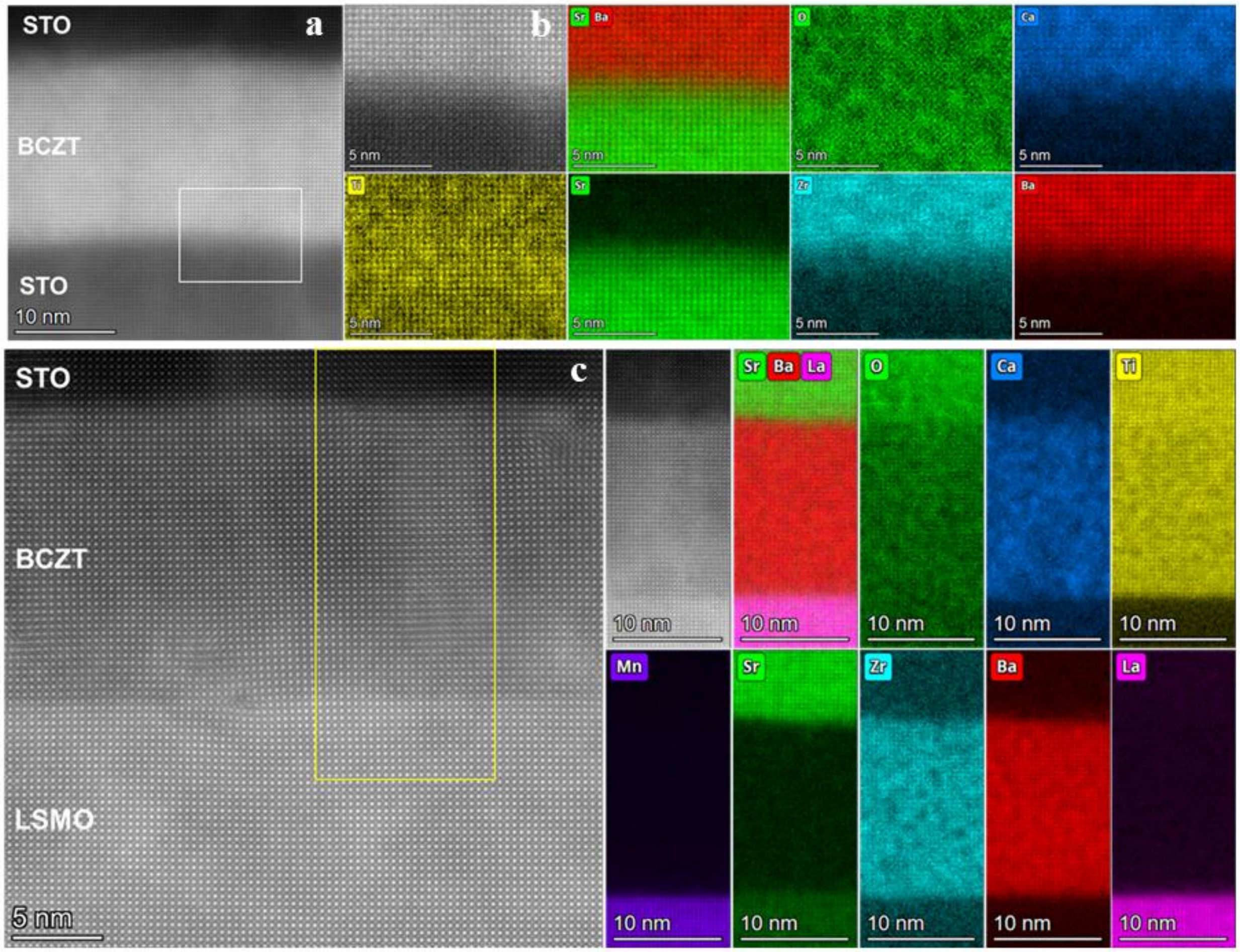

Figure S1. HAADF-STEM images and corresponding EDS elemental maps of the BCZT/STO SL and BCZT/LSMO interface. (a) Cross-sectional HAADF-STEM overview image showing the BCZT layer sandwiched between STO layers. Individual layers are labelled, highlighting the well-defined interfaces and uniform thickness. The white box marks the region shown at higher magnification. **(b)** Atomic-resolution HAADF-STEM image of the BCZT/STO interface and corresponding EDS elemental maps for Sr, Ba, O, Ca, Ti, Zr, and Ba, confirming the compositional modulation and chemical separation between BCZT and STO layers. **(c)** Cross-sectional HAADF-STEM image of the BCZT layer grown on the LSMO bottom electrode and corresponding EDS elemental maps for Sr, Ba, La, O, Ca, Ti, Mn, and Zr, demonstrating the abrupt BCZT/LSMO interface and well-defined chemical distribution of each layer.

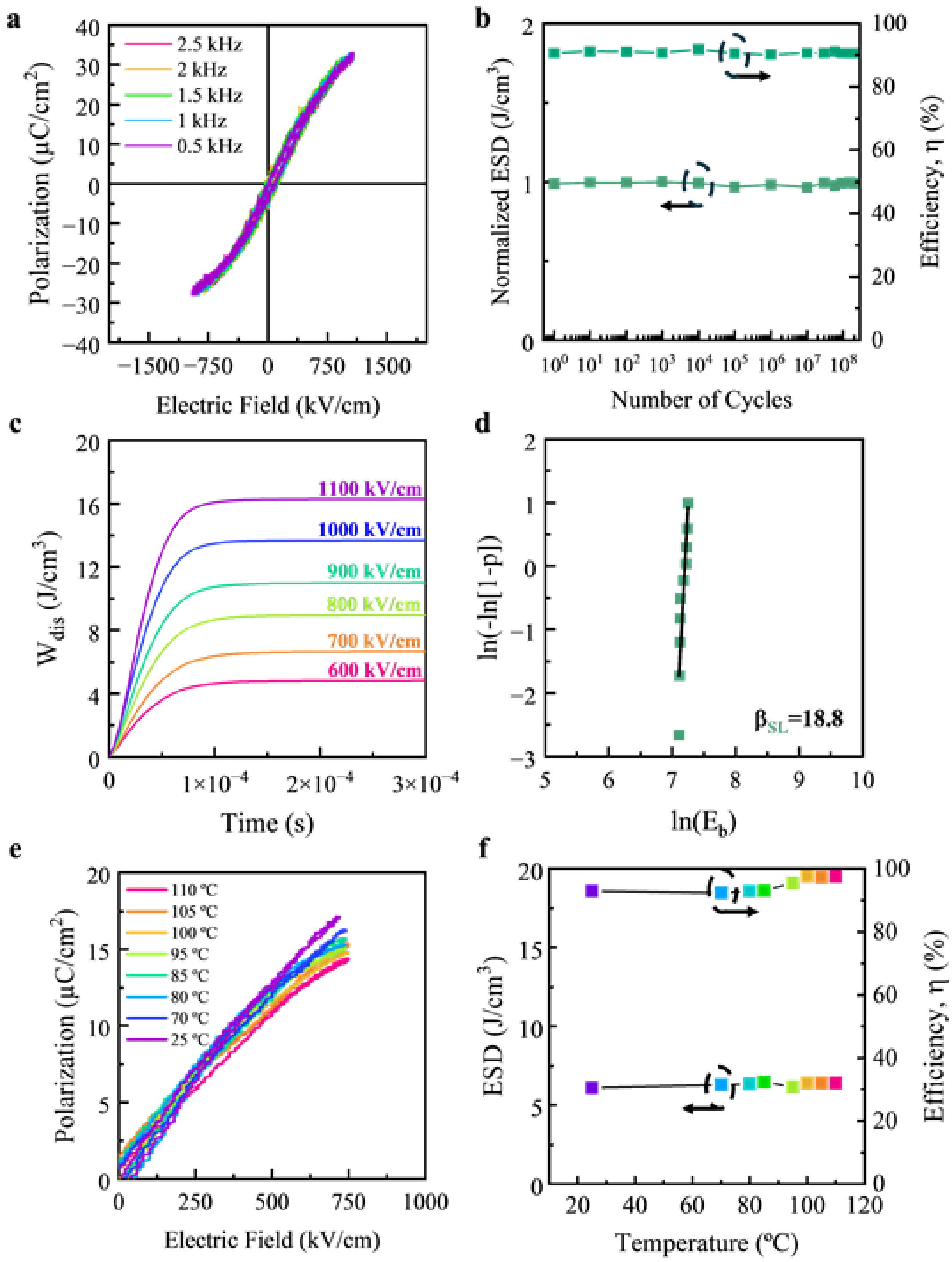

Figure S2. Stability of the ferroelectric and energy storage performance of the the BCZT/STO SL thin film capacitors. (a) Frequency dependent P-E loops. **(b)** Energy storage performance as a function of the number of cycles. **(c)** Discharge energy density as a function of time from overdamped charge-discharge measurement at electric fields from 600 to 900 kV/cm. **(d)** Weibull statistical analysis of the breakdown field. **(e)** Unipolar P-E loops and **(f)** Energy storage performance as a function of temperature.

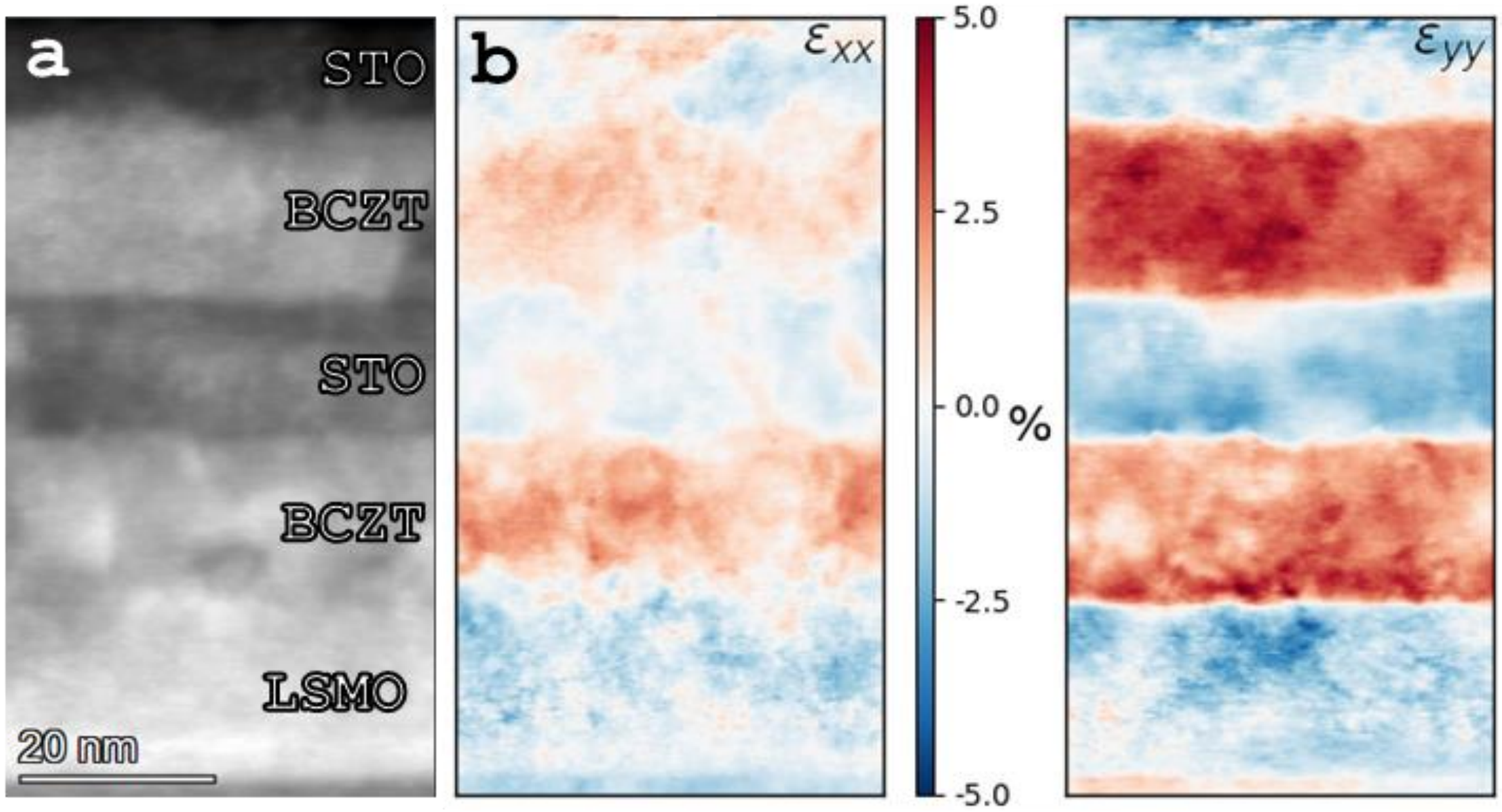

Figure S3. Structural, strain and compositional properties of the STO/BCZT/STO/BCZT initial layers on top of the bottom LSMO electrode. (a) Cross-sectional HAADF-STEM image of the STO/BCZT/STO/BCZT/LSMO heterostructure, showing the alternating ferroelectric BCZT layers sandwiched between STO layers and grown on an LSMO bottom electrode. **(b)** Corresponding in-plane and out-of-plane strain maps, ε_{xx} and ε_{yy}, obtained from 4D-STEM analysis, revealing pronounced strain variations across the BCZT layers. Positive and negative strain values are indicated by red and blue colours, respectively, with the colour scale expressed in percent.

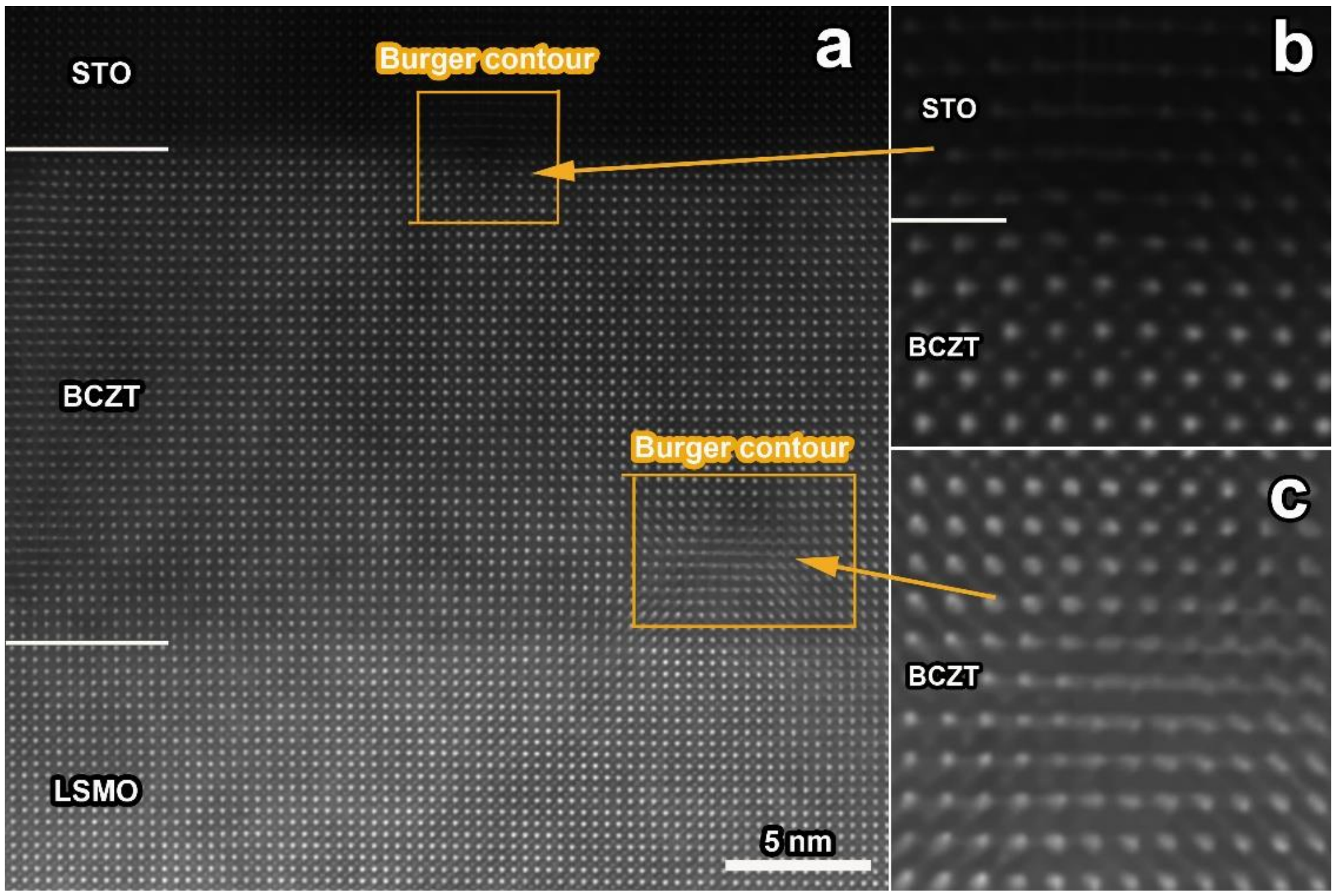

Figure S4. (a) HR-STEM image of the LSMO/BCZT/STO interfaces. Burgers circuits are drawn to highlight the misfit dislocations at both interfaces. **(b, c)** Magnified views of the regions enclosed by the Burgers circuits.

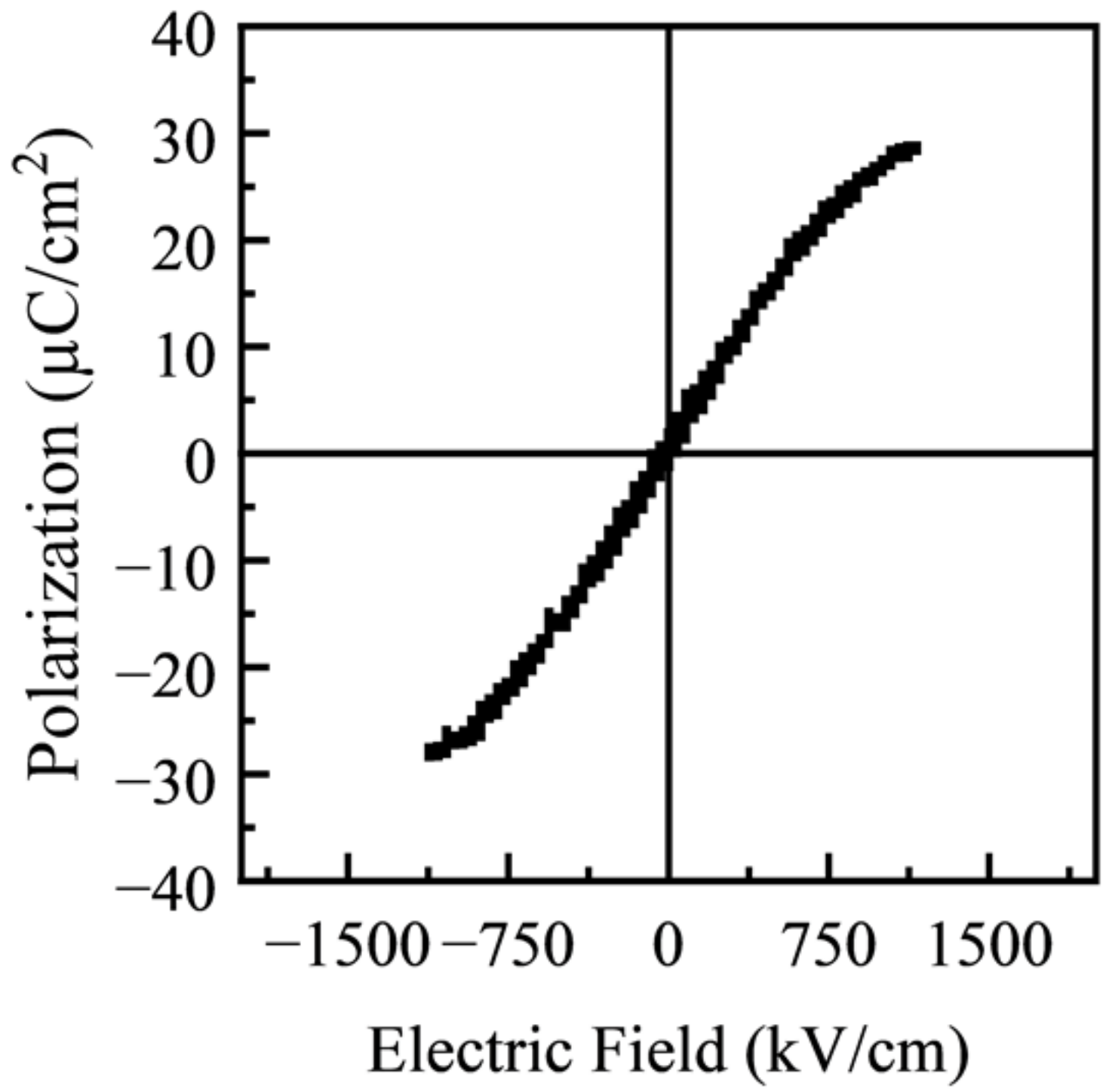

Figure S5. Polarization-electric field (P-E) hysteresis loops for a BCZT/STO SL with each individual layer has a thickness of ~3 nm.

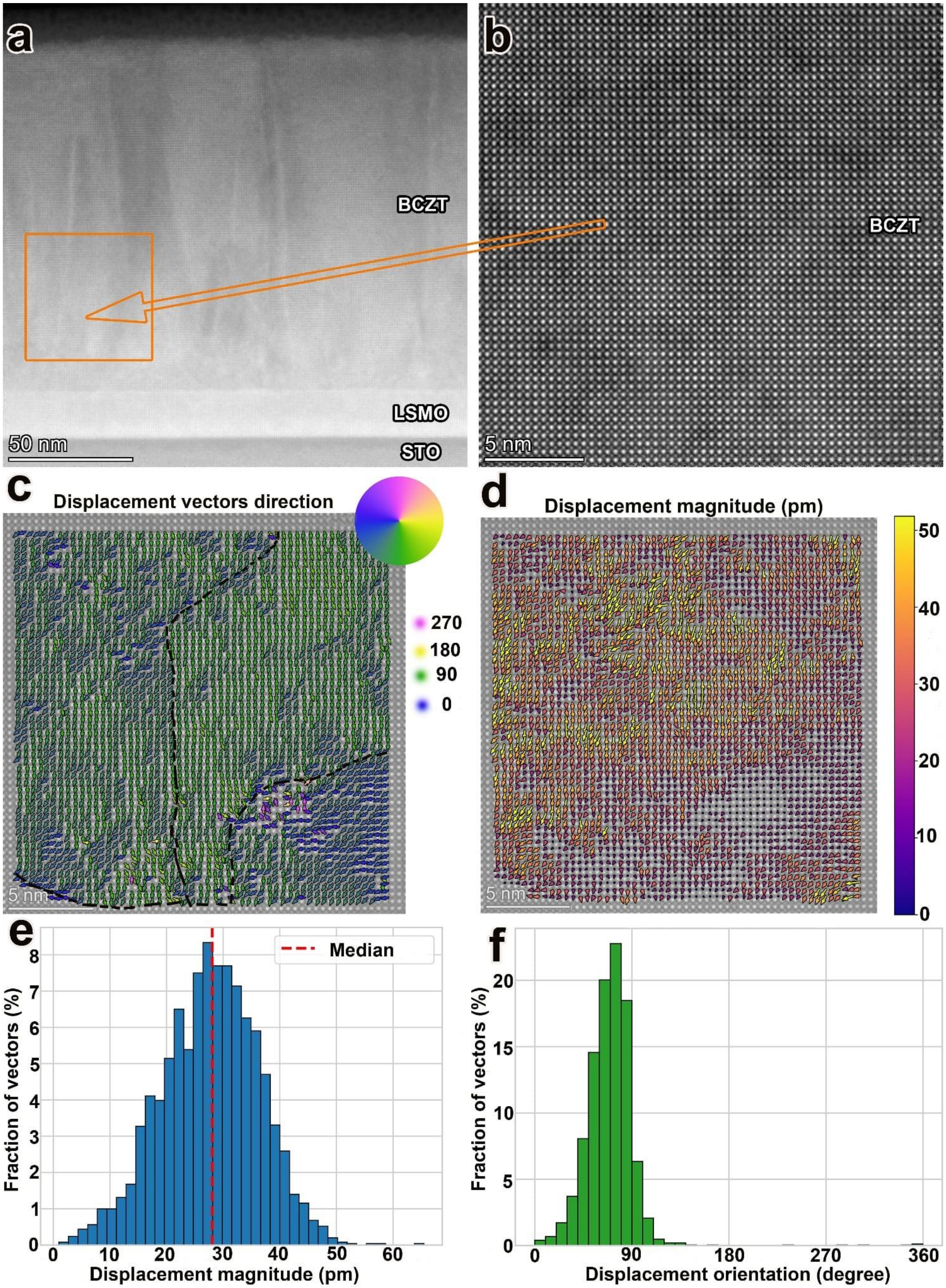
a
BCZT
LSMO
50 nm
STO
b
BCZT
5 nm
c
Displacement vectors direction
270
180
90
0
5 nm
d
Displacement magnitude (pm)
50
40
30
20
10
0
5 nm
e
Median
Fraction of vectors (%)
Displacement magnitude (pm)
f
Fraction of vectors (%)
Displacement orientation (degree)

Figure S6. Displacement-vector mapping of the BCZT/LSMO/STO heterostructure. (a) Atomic-resolution HAADF-STEM overview image of the BCZT/LSMO/STO heterostructure viewed along the [100] zone axis. **(b)** Magnified view of the BCZT area outlined by the orange square. **(c)** Displacement-vector map colored by vector orientation. **(d)** Displacement-vector map colored by displacement magnitude; black arrows serve as visual guides for the larger PNR regions. **(e)** Polarization-magnitude distribution extracted from the analyzed PZT region. **(f)** Angular distribution of the polarization vectors.

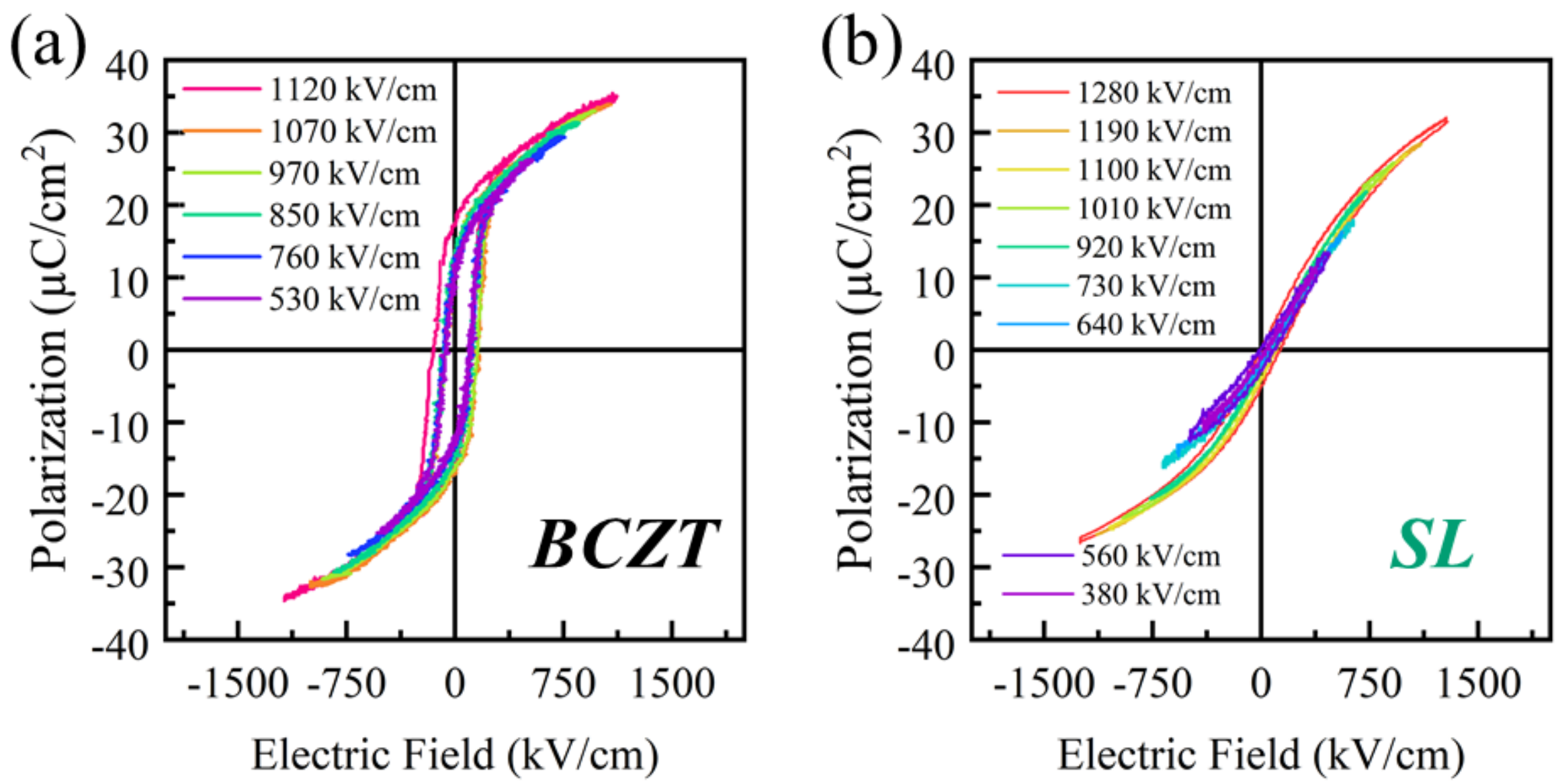

Figure S7. Polarization-electric field (P-E) hysteresis loops measured at different maximum electric fields. (a) BCZT and **(b)** the BCZT/STO SL thin film capacitors, respectively. The maximum applied electric field values are indicated in each panel.

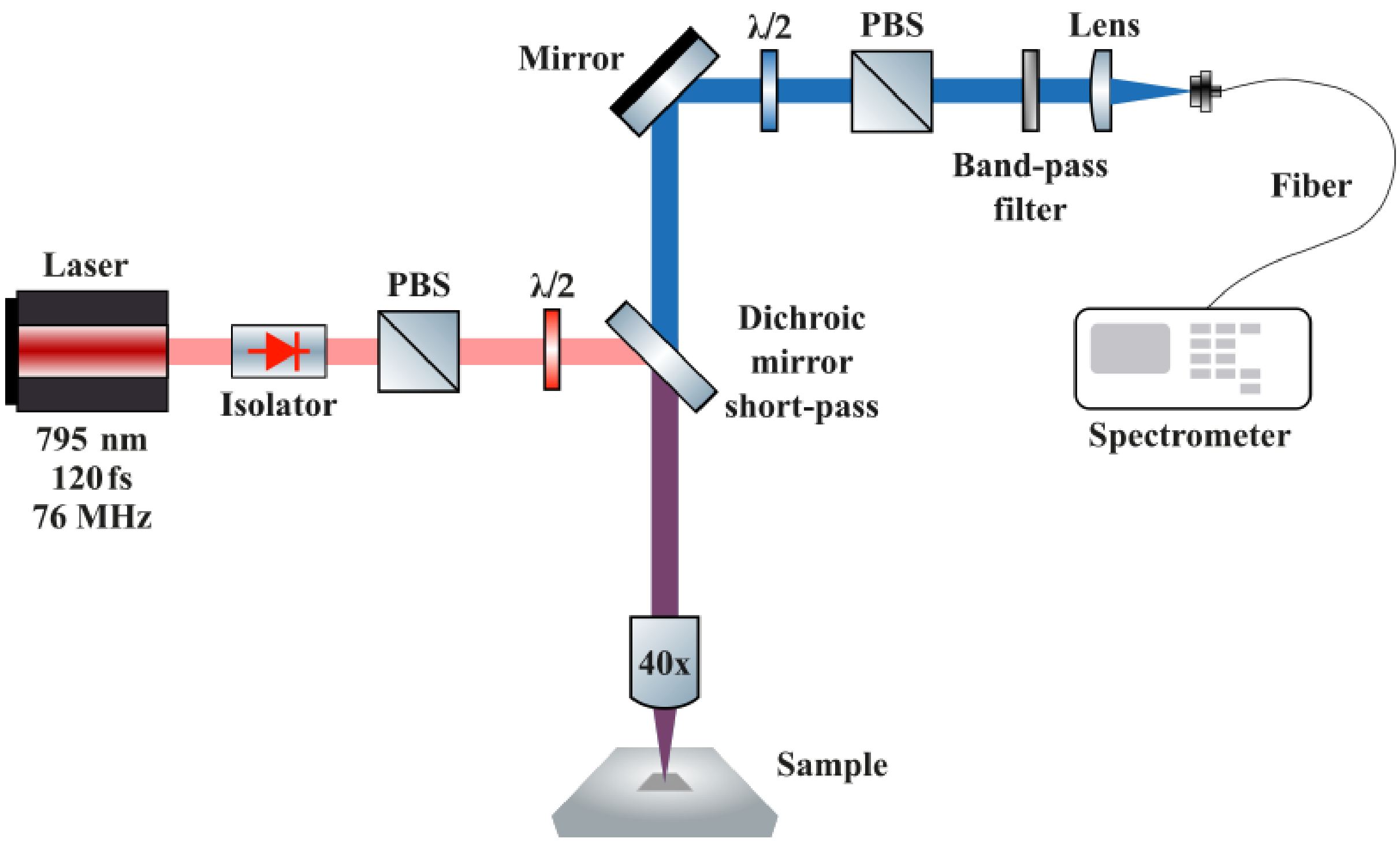

Figure S8. Experimental setup for the polarized second harmonic generation. PBS - Polarized beam splitter; λ/2 - half-waveplate.

Supplementary Table 1. Comparison of the ES performance of the BCZT thin film and BCZT/STO SL with representative lead-free FE and RFE thin films reported in the literature. The table lists the film thickness, measurement frequency, applied electric field, ESD, η, and the figure of merit U_f. The BCZT/STO SL developed in this work exhibits a markedly higher U_f compared to previously reported lead-free thin film capacitors under comparable electric fields.

Material	Thickness (nm)	Frequency (Hz)	Field (kV/cm)	ESD (J/cm^3)	Efficiency (%)	Uf (J/cm^3)	Reference
BTO-based							
BCZT/STO SL	200	1000	920	12.6	99	1723	This work
BCZT	200	1000	1070	8.9	71	31	This work
$Ba_{0.85}Ca_{0.15}Zr_{0.1}Ti_{0.9}O_3/La_{0.8}Sr_{0.2}MnO_3/Ba_{0.85}Ca_{0.15}Zr_{0.1}Ti_{0.9}O_3$	245	10000	1000	16.3	76	68	[1]
$Ba_{0.85}Ca_{0.15}Zr_{0.1}Ti_{0.9}O_3$	220	1000	1000	12.0	75	48	[2]
$BaTi_{0.98}Mn_{0.015}O_3$	150	1000	300	2.5	70	8	[3]
$Ba(Zr_{0.4}Ti_{0.6})_{0.98}Mn_{0.015}O_3$	150	1000	1500	8.0	55	18	[3]
$0.5Ba(Zr_{0.2}Ti_{0.8})O_3$-$0.5(Ba_{0.7}Ca_{0.3})TiO_3$	120	1000	1160	10.0	69	32	[4]
BNT-based							
$(0.6)Na_{0.5}Bi_{0.5}TiO_3$-$0.4Bi(Mg_{0.5}Zr_{0.5})O_3$	1000	1000	1400	37.0	60	93	[5]
$Na_{0.5}Bi_{0.5}TiO_3$	400	10000	1200	23.3	61.6	61	[6]
Fe-doped$_{0.72}Bi_{0.5}Na_{0.5}TiO_3$-$0.18Bi_{0.5}K_{0.5}TiO_3$-$0.10SrTiO_3$	900	100	1100	20.3	65.17	58	[7]
$Bi_{0.5}$ $(Na_{0.8}K_{0.2})_{0.5}TiO_3$-$0.1(BiFeO_3)$	800	1000	1200	22.1	60.85	57	[8]
$0.5Bi_{0.45}Na_{0.45}Ba_{0.1}TiO_3$-$0.5Bi(Mg_{1/3}Nb_{2/3})O_3$	200	10000	625	6.1	88.7	54	[9]
$Bi_{0.5}(Na_{0.8}K_{0.20})_{0.5}TiO_3$	300	1000	400	2.2	65	6	[10]
$(Na_{0.85}K_{0.15})_{0.5}Bi_{0.5}Ti_{0.75}Sc_{0.25}O_3$ with TiO_2 layers	860	1000	1200	22.7	51.5	47	[11]

$0.95[0.6Bi_{0.5}(Na_{0.8}K_{0.2})_{0.5}TiO_3$-$0.4SrTiO_3]$-$0.05LaAlO_3$:Mn	232	1000	1500	34.3	70.1	115	[12]
$(Na_{0.5}Bi_{0.5})TiO_3$	400	10000	1400	24.6	72	88	[13]
$0.5(Na_{0.5}Bi_{0.5})TiO_3$-$0.5Bi(Zn_{0.5}Zr_{0.5})O_3$	700	-	1500	40.8	65	117	[14]
BFO-based							
$0.68BiFeO_3$-$0.32BaTiO_3$	200	1000	900	19.0	51	39	[15]
Aurivillius-based							
$BaBi_4Ti_4O_{15}$	100	-	1500	32.5	80	163	[16]
$Ba_2Bi_4Ti_5O_{18}$	1000	-	1600	22.6	71.3	79	[17]
$Bi_6Ti_3FeAlO_{18}$	600	20000	1500	67.5	75.5	276	[18]
$0.9Sr_{0.7}Bi_{0.2}TiO_3$-$0.1BaTiO_3$-Mn	270	1000	1200	55.0	80.9	288	[19]
KNN-based							
$Na_{0.5}K_{0.5}NbO_3/BiMnO_3$	-	-	985.66	14.8	79.79	73	[20]
$0.95(K_{0.49}Na_{0.49}Li_{0.02})(Nb_{0.8}Ta_{0.2})O_3$- $0.05CaZrO_3$-0.04Mn	186		1760	26.9	64.3	75	[21]
$K_{0.5}Na_{0.5}Nb_{0.96}Mn_{0.04}O_3$	300	1000	1280	12.6	60.2	32	[22]

References

[1] Lakouader, A. et al. BCZT/LSMO/BCZT sandwich film: Toward high-temperature energy storage capacitors. Materialia 38, 102309 (2024).

[2] Lakouader, A. et al. Thermally stable electrostatic energy storage in X9R-Type $BaTiO_3$-based thin films elaborated by chemical solution deposition. J. Energy Storage 141, 119421 (2026).

[3] Konsago, S. W. et al. Engineering the Microstructure and Functional Properties of $0.5Ba(Zr_{0.2}Ti_{0.8})O_3$-$0.5(Ba_{0.7}Ca_{0.3})TiO_3$ Thin Films. ACS Appl. Electron. Mater. 6, 4467-4477 (2024).

[4] Angermann, M. et al. High energy density and reliable $BaZr_xTi_{1-x}O_3$ thin film capacitors from novel aqueous chemical solution deposition. J. Eur. Ceram. Soc. 45, 117465 (2025).

[5] Luo, X. et al. Flexible lead-free $Na_{0.5}Bi_{0.5}TiO_3$-based film capacitor with stable energy storage performances. J. Mater. Sci. Mater. Electron. 34, 604 (2023).

[6] Wang, F., Zhu, C. & Zhao, S. Good energy storage properties of $Na_{0.5}Bi_{0.5}TiO_3$ thin films. J. Alloys Compd. 869, 159366 (2021).

[7] Wu, S. et al. Enhanced piezoelectricity and energy storage performances of Fe-doped BNT-BKT-ST thin films. Ceram. Int. 44, 21289-21294 (2018).

[8] Chen, P. et al. Enhanced dielectric and energy-storage properties in $BiFeO_3$-modified $Bi_{0.5}(Na_{0.8}K_{0.2})_{0.5}TiO_3$ thin films. Ceram. Int. 43, 13371-13376 (2017).

[9] Wu, J. et al. Excellent Energy Storage and Photovoltaic Performances in $Bi_{0.45}Na_{0.45}Ba_{0.1}TiO_3$-Based Lead-Free Ferroelectricity Thin Film. Ceramics 7, 1043-1052 (2024).

[10] Quan, N. D., Toan, T. Q. & Hung, V. N. Influence of Crystallization Time on Energy-Storage Density and Efficiency of Lead-Free $Bi_{0.5}(Na_{0.8}K_{0.2})_{0.5}TiO_3$ Thin Films. Adv. Condens. Matter Phys. 2019, 4949365 (2019).

[11] Wu, Y., Hu, Y., Wang, X., Zhong, C. & Li, L. Temperature- and frequency-dependent dielectric response and energy-storage performance in high (100)-oriented Sc-doped $(Na_{0.85}K_{0.15})_{0.5}Bi_{0.5}TiO_3$ films. RSC Adv. 7, 51485-51494 (2017).

[12] Sun, M. et al. Enhanced Energy Storage Performance in BNKT-Based Lead-Free Thin Films at Low Electric Fields. ACS Appl. Electron. Mater. 7, 11284-11293 (2025).

[13] Liu, X., Yao, Y., Wang, X., Zhao, L. & San, X. Energy Storage Performance of $(Na_{0.5}Bi_{0.5})TiO_3$ Relaxor Ferroelectric Film. Coatings 14, 801 (2024).

[14] Sun, N., Li, Y., Liu, X. & Hao, X. High energy-storage density under low electric field in lead-free relaxor ferroelectric film based on synergistic effect of multiple polar structures. J. Power Sources 448, 227457 (2020).

[15] Hu, Y. et al. High energy storage performance in lead-free $BiFeO_3$-$BaTiO_3$ ferroelectric thin film fabricated by pulsed laser deposition. AIP Adv. 9, 085005 (2019).

[16] Keum, Y. H., Shin, H. W. & Son, J. Y. Improvement of energy storage performance by controlling the crystallinity of Aurivillius $BaBi_4Ti_4O_{15}$ thin films. J. Korean Ceram. Soc. 60, 959-966 (2023).

[17] Song, D. & Yang, J. Lead-free $Ba_2Bi_4Ti_5O_{18}$ thin film capacitors for energy storage applications. Results Phys. 13, 102175 (2019).

[18] Liu, Q. et al. Intercalation strategy induced superior energy storage performance in Aurivillius $Bi_6Ti_3FeAlO_{18}$ film under low and medium electric fields. J. Materiomics 12, 101113 (2026).

[19] Zhou, J. et al. Optimized energy storage performance of SBT-based lead-free relaxor ferroelectric thin film. J. Mater. Sci. Mater. Electron. 34, 1294 (2023).

[20] Sun, Y., Zhou, Y., Lu, Q. & Zhao, S. High Energy Storage Efficiency with Fatigue Resistance and Thermal Stability in Lead-Free $Na_{0.5}K_{0.5}NbO_3$/$BiMnO_3$ Solid-Solution Films. Phys. Status Solidi RRL 12, 1700364 (2018).

[21] Cheng, Y.-Y.-S. et al. All-Inorganic Flexible (K, Na)NbO_3-Based Lead-Free Piezoelectric Thin Films Spin-Coated on Metallic Foils. ACS Appl. Mater. Interfaces 13, 39633-39640 (2021).

[22] Zhao, C., Meng, X., Wang, W. & Zhou, Y. Energy storage performance of (K, Na)NbO_3 ferroelectric thin films with Mn-Ta and Mn-Ti co-doping. Ceram. Int. 45, 13772-13779 (2019).
