# Supplementary material for: Superlattice Engineering in Relaxor Ferroelectric-based Capacitors for Ultra-Highly Efficient Recoverable Energy Storage Density
Source: Nat Commun. 2026 Oct 5;17:11338. doi: 10.1038/s41467-026-77971-6 (PMC13728193; doi:10.1038/s41467-026-77971-6)
Supplement: Supplementary file 2 [file 41467_2026_77971_MOESM2_ESM.pdf]

nature portfolio

Peer Review File

Superlattice Engineering in Relaxor Ferroelectric-based Capacitors for Ultra-Highly Efficient Recoverable Energy Storage Density

Corresponding Author: Dr José Silva

This file contains all reviewer reports in order by version, followed by all author rebuttals in order by version.

Version 0:

Reviewer comments:

Reviewer #1

(Remarks to the Author)
In this paper, the authors reported "Superlattice Engineering in Relaxor Ferroelectric-based Capacitors for Ultra-Highly Efficient Recoverable Energy Storage Density", exploring how superlattice engineering enhances the energy storage performance of BCZT/STO multilayer films under low electric fields. According to the manuscript, I did not find the enough novelty and innovative of the current work to be published in Nature Communications. I suggest the authors consider the following points.
1. The imprint effect and built-in electric field mentioned in this article should be further explored and discussed. The author should clarify which of the three factors, asymmetric electrode, interface effect, and substrate stress, is the key factor causing imprinting effect.
2. Why is the loss of SL greater than that of BCZT in Figures 3 (d) and (e).
3. In Figure 4 (g), we can observe a high proportion of 225 degree polarization angles, while in Figure 4 (c), almost all polarization vectors are labeled as vertical and parallel directions. The author should explain this point carefully. Importantly, the author mentioned that the BCZT component is located in morphotropic phase boundary.
4. In this work, the ESD reaches 20.4 J/cm3 at the highest applied field of 1280 kV/cm, with an η value of 86%. The energy storage density is much lower than the currently reported energy storage density of thin film capacitors. This seriously weakens the innovation and uniqueness of this work.
5. In lines 334 and 335, the author mentions 'In addition, the BCZT/STO SL capacitor demonstrate a stable ES performance, up to 2×108 cycles (data not shown here)'. However, this data is not seen in both the manuscript and supplementary materials. Why? In addition, besides fatigue testing, the author should supplement frequency stability, temperature stability, and charge-discharge testing.

Reviewer #2

(Remarks to the Author)
This manuscript presents an innovative approach to enhancing energy storage performance in lead-free relaxor ferroelectric capacitors through superlattice (SL) engineering, combining BCZT and STO layers with asymmetric electrodes (LSMO/Au). However, the manuscript suffers from several issues that undermine the strength of its claims as follows:
1. The manuscript reports ESD = 12.6 J/cm³ at 920 kV/cm and 20.4 J/cm³ at 1280 kV/cm for the BCZT/STO SL. However, these values do not surpass recently published results in lead-free systems such as Nat. Commun. 16, 886 (2025) and Nat. Commun. 16, 1300 (2025). The manuscript's primary performance advantage appears to be the ultra-high efficiency (η ~ 99%) rather than ESD. However, this extreme efficiency value is highly sensitive to measurement uncertainty.
2. The manuscript repeatedly emphasizes "low applied electric field" as a key advantage. Therefore, the authors should provide a clear quantitative definition of what constitutes a "low electric field" in the context of thin film capacitors, with reference to the practical voltage requirements of target applications. Furthermore, the ESD normalized by electric field should be compared with other relevant reported references.
3. The manuscript attributes the observed imprint effect in the P-E hysteresis loops entirely to the work function difference between the LSMO bottom electrode and the Au top electrode. However, imprint in ferroelectric thin films has multiple possible origins. The manuscript provides no control experiments to isolate the electrode asymmetry effect, nor does it present leakage current or interface charge distribution measurements to verify the work function hypothesis.

4. The figure of merit Uf = ESD/(1 − η) is numerically unstable near η → 100%, and the claimed "5× higher" comparison is unreliable. The authors should explicitly discuss the sensitivity of Uf to η measurement uncertainty and provide error propagation analysis.
5. The manuscript claims that "a higher breakdown electric field is obtained in the BCZT/STO SL compared to the BCZT film" but provides no Weibull statistical analysis and no information on the number of samples tested for breakdown.
6. The manuscript's central claim is supported only by static HAADF-STEM observations. This is insufficient to establish a causal relationship between the SL structure and the observed polar nanoregion configuration. Theoretical calculations such as phase-field simulations are necessary to supplement the experimental observations and analyze the relationship between SL structure and polar nanodomain formation.

Version 1:

Reviewer comments:

Reviewer #1

(Remarks to the Author)
The comments have been well addressed. It can be considered for publication in Nature Communications.

Reviewer #2

(Remarks to the Author)
Although the have responded constructively to several concerns, the most critical issue remain inadequately addressed. The original concern was that static HAADF-STEM images cannot establish a causal mechanism linking the superlattice structure to polar nanoregion behavior and energy-storage performance. The requested phase-field simulation (or equivalent GLD-based modeling) was not performed, with the authors stating it is “beyond the scope of this revision”.
The phase-field studies cited (BTO/STO, BFO/STO) concern polar topological structures, not polar nanoregions. Without theoretical modeling, the paper’s central claim that SL governs polar nanoregions formation remains unsubstantiated.

Version 2:

Reviewer comments:

Reviewer #2

(Remarks to the Author)
The authors have thoroughly addressed the comments. Accordingly, I recommend this paper for acceptance.

Open Access This Peer Review File is licensed under a Creative Commons Attribution 4.0 International License, which

permits use, sharing, adaptation, distribution and reproduction in any medium or format, as long as you give appropriate credit to the original author(s) and the source, provide a link to the Creative Commons license, and indicate if changes were made.
In cases where reviewers are anonymous, credit should be given to 'Anonymous Referee' and the source.
The images or other third party material in this Peer Review File are included in the article's Creative Commons license, unless indicated otherwise in a credit line to the material. If material is not included in the article's Creative Commons license and your intended use is not permitted by statutory regulation or exceeds the permitted use, you will need to obtain permission directly from the copyright holder.
To view a copy of this license, visit https://creativecommons.org/licenses/by/4.0/

Response Letter

Dear Editor,

Thank you very much for expediting the review of our manuscript titled "Superlattice Engineering in Relaxor Ferroelectric-based Capacitors for Ultra-Highly Efficient Recoverable Energy Storage Density" (Manuscript Ref.: NCOMMS-26-027279). We appreciate the comments and suggestions from the Reviewers and are thankful to them for their invaluable time and effort in evaluating our work. As per the Reviewers' comments, we have revised the manuscript incorporating all their suggestions. The modifications to the manuscript are highlighted in blue color font in the revised file. Specific responses to the Reviewer's comments are listed below.

Please do let us know if any additional information is needed. Thank you.

Sincerely,
Dr. José Silva
University of Minho

Reviewer comments and author responses:

Reviewer #1:

In this paper, the authors reported "Superlattice Engineering in Relaxor Ferroelectric-based Capacitors for Ultra-Highly Efficient Recoverable Energy Storage Density", exploring how superlattice engineering enhances the energy storage performance of BCZT/STO multilayer films under low electric fields. According to the manuscript, I did not find the enough novelty and innovative of the current work to be published in Nature Communications. I suggest the authors consider the following points.

We thank the Reviewer for the critical reading of our manuscript. We have addressed the concern regarding novelty by making this clearer, and also by substantially expanding the manuscript with new data as explained in detail below.

1. The imprint effect and built-in electric field mentioned in this article should be further explored and discussed. The author should clarify which of the three factors, asymmetric electrode, interface effect, and substrate stress, is the key factor causing imprinting effect.

A: We thank the reviewer for this important comment. To understand the origin of the imprint effect, we have investigated the structural, strain and compositional properties of

the STO/BCZT/STO/BCZT initial layers on top of the bottom LSMO electrode. Thus 4D-STEM experiments were conducted. The 4D-STEM data have been acquired on the HAADF-STEM image presented in Fig S3(a).

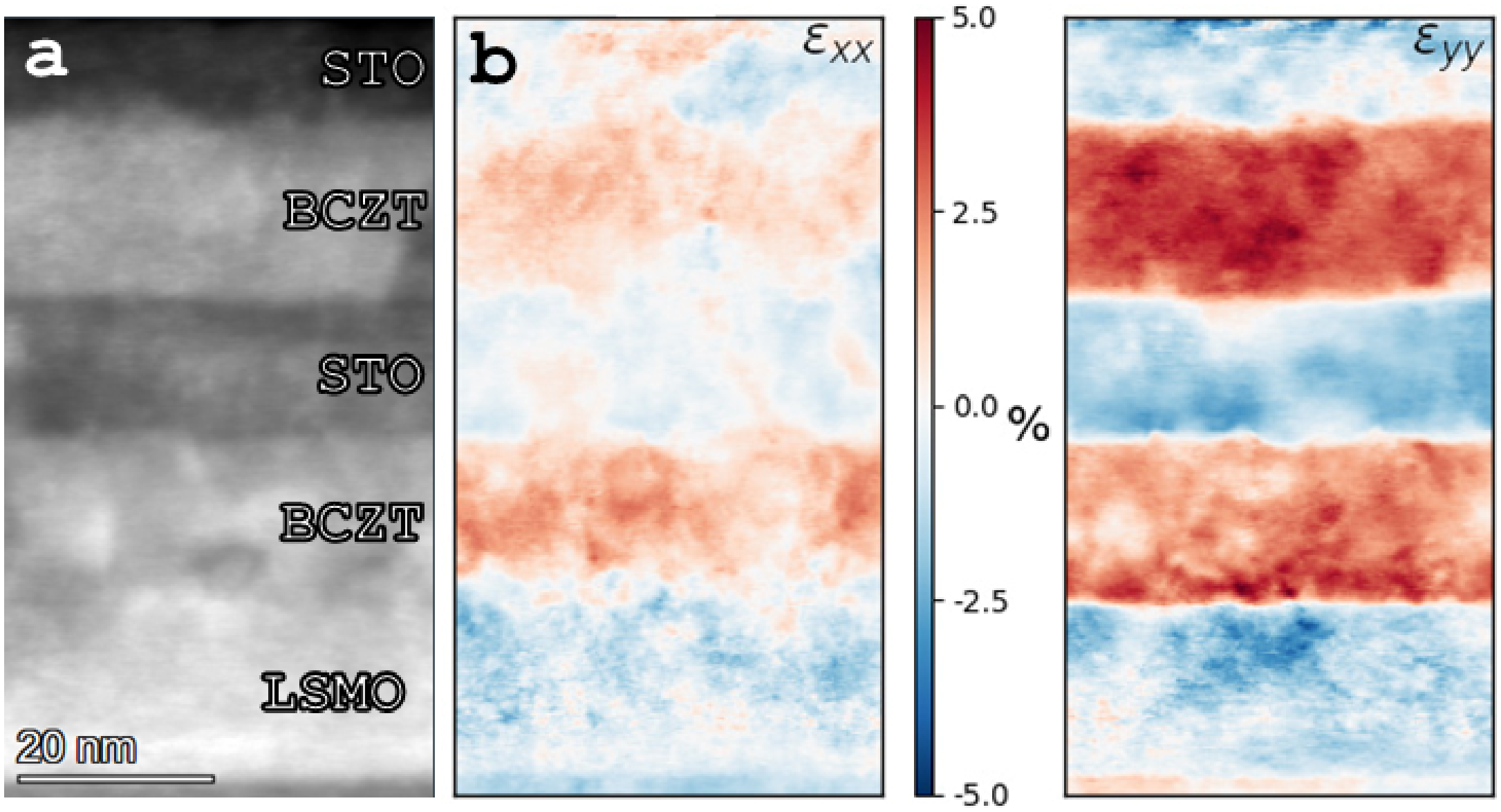

Figure S3. Structural, strain and compositional properties of the STO/BCZT/STO/BCZT initial layers on top of the bottom LSMO electrode. (a) Cross-sectional HAADF-STEM image of the STO/BCZT/STO/BCZT/LSMO heterostructure, showing the alternating ferroelectric BCZT layers sandwiched between STO layers and grown on an LSMO bottom electrode. **(b)** Corresponding in-plane and out-of-plane strain maps, ε_{xx} and ε_{yy}, obtained from 4D-STEM analysis, revealing pronounced strain variations across the BCZT layers. Positive and negative strain values are indicated by red and blue colours, respectively, with the colour scale expressed in percent.

The corresponding 4D-STEM/ACOM maps in Fig. S3(b,c) were obtained using a tetragonal $BaTiO_3$-type perovskite structure as the crystallographic indexing model. Since the exact unstrained lattice parameters and room-temperature symmetry of the present BCZT composition are not independently known, these maps are interpreted primarily as relative spatial variations of the local lattice spacing rather than as an absolute quantitative determination of elastic strain. In addition, the values displayed within the STO layers are not quantitatively interpreted using the BCZT/$BaTiO_3$-type reference.

The most reproducible feature of the 4D-STEM analysis is the presence of an anisotropic and depth-dependent lattice distortion within the BCZT layers. The out-of-plane component exhibits a stronger variation, particularly in the lower BCZT layer close to the LSMO electrode, whereas the in-plane component is weaker and spatially heterogeneous. The magnitude of the lattice distortion changes over the first few nanometers from the BCZT/LSMO interface, indicating the presence of an interface-related structural gradient rather than a homogeneous biaxial strain state.

We note that the absolute magnitude of the mapped values may also be influenced by the choice of the reference lattice, local specimen thickness, residual preparation-induced surface damage, specimen bending or tilt, dynamical diffraction and uncertainties in the fitting of the Bragg-disk positions. The cross-sectional specimen was prepared by conventional mechanical thinning and ion milling, and the different thinning rates of LSMO, BCZT and STO may produce local thickness and surface-damage variations. Therefore, the maps are used to identify robust spatial trends in the lattice distortion, rather than to assign exact absolute strain values.

To further examine the mechanisms responsible for the relaxation of the interfacial distortion, high-resolution STEM images were analyzed at the BCZT/STO interfaces and within the BCZT layers. Burgers circuits constructed around representative lattice defects fail to close, confirming the presence of dislocations with non-zero Burgers vectors, as shown in Fig. S4. These defects can provide a mechanism for partial accommodation of the lattice mismatch and may contribute to the observed local relaxation. However, we recognize that differences in the boundary conditions of the successive BCZT layers, point-defect distributions and local specimen-thickness effects may also influence the measured lattice parameters. Accordingly, dislocation formation is considered to be an important contribution to the relaxation process, rather than the exclusive origin.

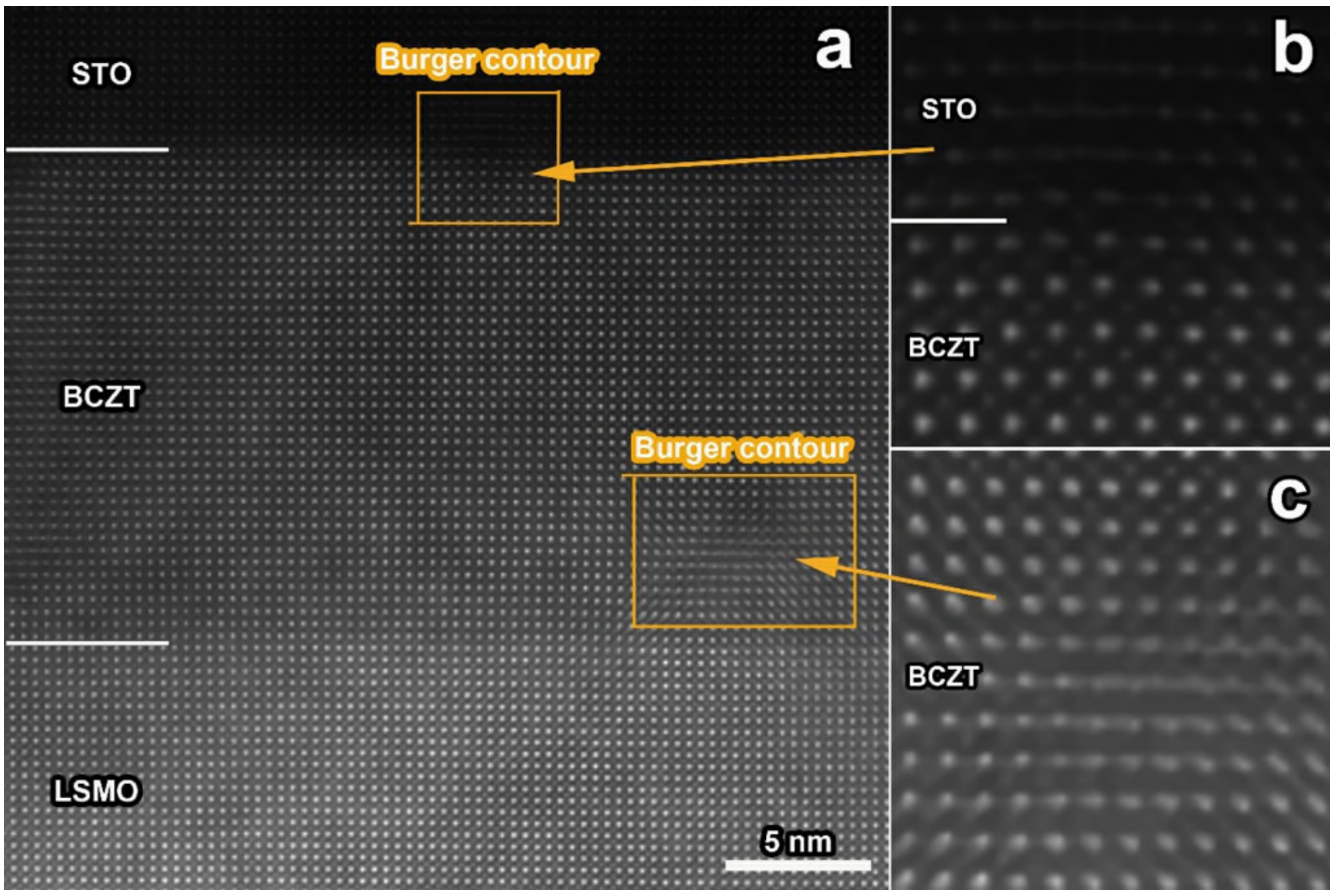

Figure S4. (a) HR-STEM image of the LSMO/BCZT/STO interfaces. Burgers circuits are drawn to highlight the misfit dislocations at both interfaces. **(b, c)** Magnified views of the regions enclosed by the Burgers circuits.

Furthermore, STEM-EDS elemental maps (Fig. 2 and S1) confirm the multilayer architecture and demonstrate clear elemental partitioning, with Ba, Ca and Zr concentrated within the BCZT layers, Sr and Ti associated with STO, and La and Mn localized within the LSMO electrode. Therefore, composition gradients [V. P. Afanasjev, A. A. Petrov, I. P. Pronin, E. A. Tarakanov, E. J. Kaptelov and J. Graul, J. Phys.: Condens. Matter, 2001, 13, 8755–8763.] can be excluded as the driving mechanism for the imprint effect, with the strain gradient [B. C. Jeon, D. Lee, M. H. Lee, S. M. Yang, S. C. Chae, T. K. Song, S. D. Bu, J.-S. Chung, J.-G. Yoon and T. W. Noh, Adv. Mater., 2013, 25, 5643–5649.] instead of being the dominant contributor.

In order to further confirm if the strain gradient, after 2-3 nm across the BCZT layer is responsible for the observed imprint effect, we have measured the P-E loops for a BCZT/STO SL with each individual layer being of thickness ~3 nm. Figure S5 shows the P-E loop and apart from the slim P-E loops typical of the RFE behaviour, the imprint is no longer observed. Therefore, we conclude that a substrate-induced strain gradient arising from the lattice mismatch at the BCZT/LSMO interface is the primary factor

responsible for the imprint effect, while the asymmetric electrode contribution is a negligible effect.

This is included in the manuscript on pages no. 10 and 11.

2. Why is the loss of SL greater than that of BCZT in Figures 3 (d) and (e).

A: The dielectric loss is higher in the BCZT/STO SL compared to BCZT thin films primarily due to the increased density of interfaces, which create strong depolarizing fields (see: Nano Lett. 2012, 12, 6, 2846–2851). In addition, increasing numbers of interfaces may increase the number of defects that results in leakage that causes a greater loss.

This is included in the manuscript on pages no. 12 and 13.

3. In Figure 4 (g), we can observe a high proportion of 225 degree polarization angles, while in Figure 4 (c), almost all polarization vectors are labeled as vertical and parallel directions. The author should explain this point carefully. Importantly, the author mentioned that the BCZT component is located in morphotropic phase boundary.

A: We thank the Reviewer for this important observation. This apparent discrepancy originates from the simplified labeling used in the original polarization color-coded vector map in Fig. 4(c). In the initial version, to avoid overloading the figure, only the main vertical and in-plane/parallel polarization directions were explicitly indicated. However, the domains with polarization angle close to 225° were already present in the color-coded vector map and were mentioned in the manuscript, although they were not explicitly labeled in the figure. Therefore, to avoid misinterpretation, we have modified Fig. 4(c) by explicitly including the 225° polarization direction in the legend/annotation of the color-coded vector map.

This observation is consistent with the localization of the BCZT component near the morphotropic phase boundary (MPB), where multiple polarization states and easy polarization rotation pathways can coexist. Thus, the presence of oblique polarization directions is not unexpected, but rather supports the complex polarization landscape associated with the MPB composition.

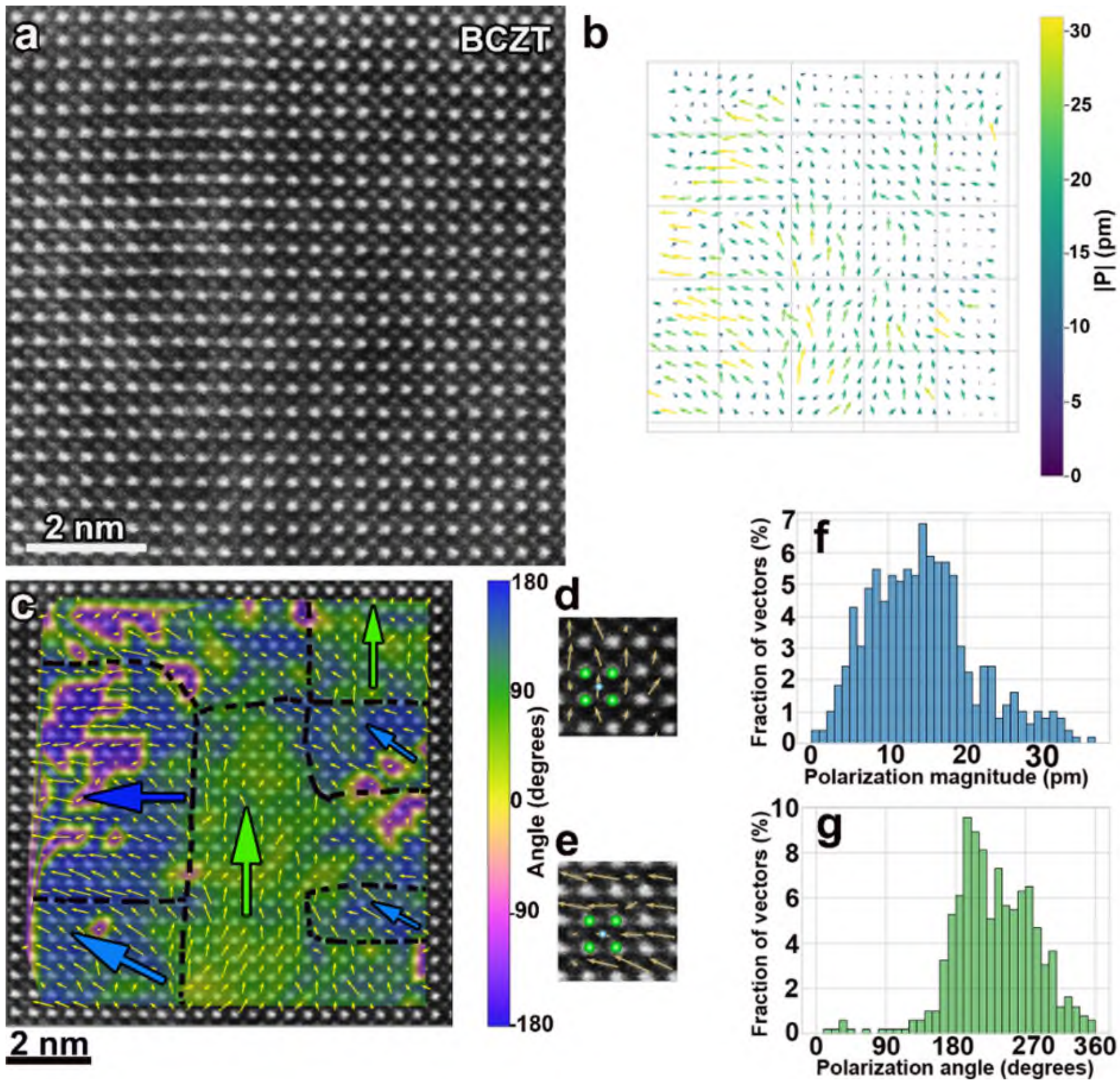

Figure 4. (a) Atomic resolution HAADF-STEM image of the BCZT layer in the SL. **(b)** Polarization vector map colored by magnitude. **(c)** Atomic-resolution HAADF-STEM polarization vector images along [100]c direction colored by their direction. Yellow arrows show the displacement of each B-site cation inside the unit cell. PNRs with the same B-site cation displacement vectors are circled as nanodomains using dark dashed lines. **(d)-(e)** Magnified images of selected areas from (c) with the BCZT unit cell overlapped. **(f)-(g)** Polarization vector magnitude and direction distribution of B-site cations obtained from the HAADF-STEM image (c).

4. In this work, the ESD reaches 20.4 J/cm^3 at the highest applied field of 1280 kV/cm, with an η value of 86%. The energy storage density is much lower than the currently reported energy storage density of thin film capacitors. This seriously weakens the innovation and uniqueness of this work.

A: Typically, the much higher ESD values in perovskite RFEs and FEs thin films for ES are achieved at **impractically high electric fields** (>3 MV/cm) [1, 12-16]. For instance, M. D. Nguyen et al. demonstrated that RFE/PE multilayered devices combining PE-like $Ba_{0.6}Sr_{0.4}TiO_3$ with RFE $BaZr_{0.4}Ti_{0.6}O_3$ (BZT) layers on $SrTiO_3$-buffered Si substrates

can exhibit a large ESD of ≈165.6 J/cm^3, with an ŋ of 93 %, due to the extremely large breakdown field of 7.5 MV/cm [10]. However, high ESD values in relaxor ferroelectric thin films at moderate operation voltage of <50 V corresponding to **applied electric fields of ~500-2500 kV/cm are crucial for developing highly-efficient ES systems**, to feed micro-electromechanical systems (MEMS), or sensors.

This is included in the manuscript on page no. 3.

For this reason, in Fig 5(b)-(d) we have compared the performance of our capacitors with the ones reported on literature in the field range 500-2000 kV/cm. While the ESD is comparable to most of the works reported in the literature, especially the ones based on $BaTiO_3$, **the efficiency reported here is much higher**. Therefore, our central claim is not the highest absolute ESD, but the **combination of high ESD at a low-to-moderate field/voltages with ultra-high efficiency (η ≈ 99%), which is the relevant figure of merit for low-voltage, miniaturized applications.**

5. In lines 334 and 335, the author mentions 'In addition, the BCZT/STO SL capacitor demonstrate a stable ES performance, up to 2×10^8 cycles (data not shown here)'. However, this data is not seen in both the manuscript and supplementary materials. Why? In addition, besides fatigue testing, the author should supplement frequency stability, temperature stability, and charge-discharge testing.

A: We thank the Reviewer for these valuable suggestions. In the revised manuscript, we have now provided the results related to frequency stability, temperature stability, endurance and charge-discharge testing. Thus, in Fig. S2(a) we show the frequency-dependent P-E measurements performed from 0.5 to 2.5 kHz, under an applied electric field of 1100 kV/cm. The P-E loops remain slim and nearly overlapping throughout the investigated frequency range, confirming that the response is intrinsic.

This is included in the manuscript on page no. 10.

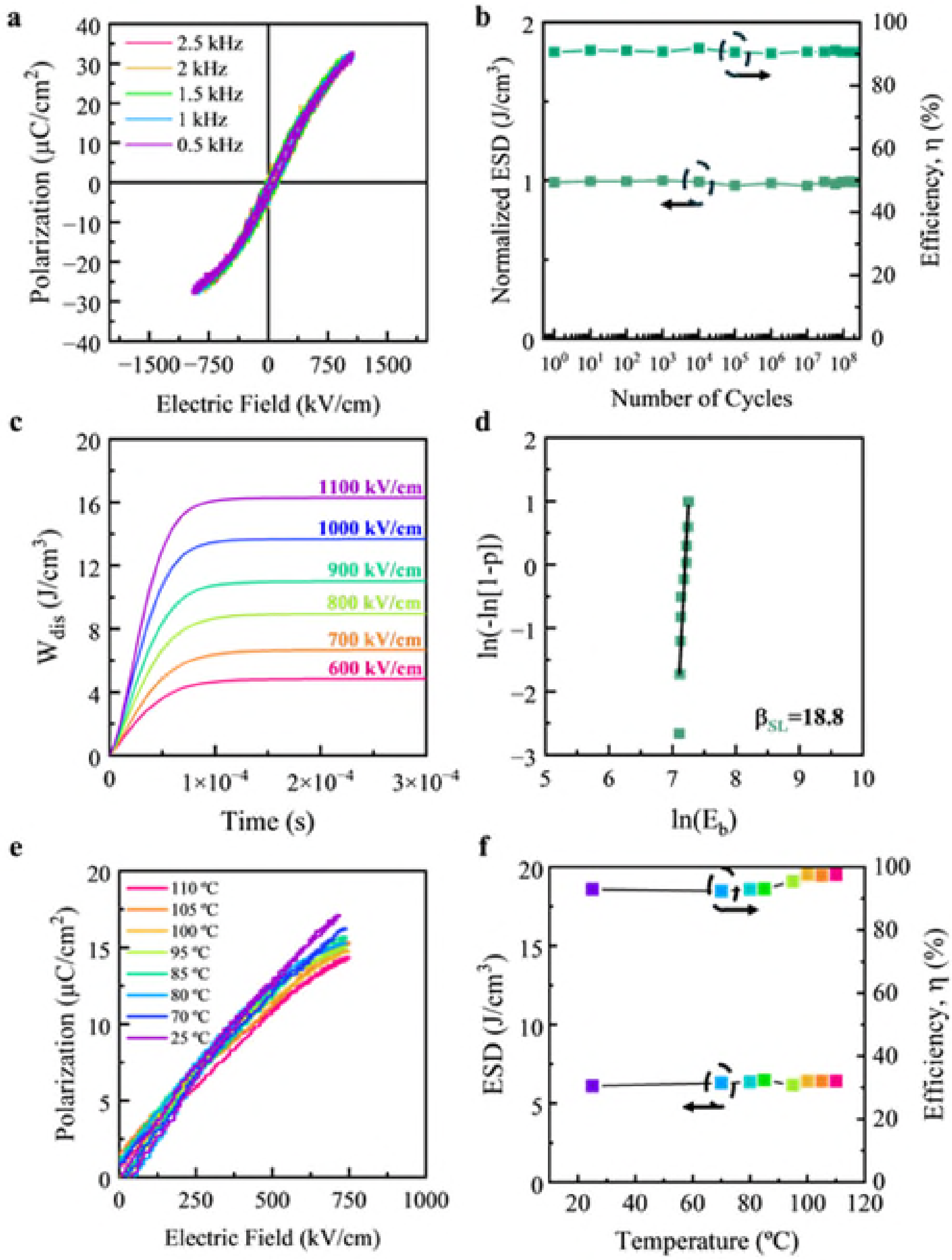

Figure S2. Stability of the ferroelectric and energy storage performance of the the BCZT/STO SL thin film capacitors. (a) Frequency dependent P-E loops. **(b)** Energy storage performance as a function of the number of cycles. **(c)** Discharge energy density as a function of time from overdamped charge-discharge measurement at electric fields from 600 to 900 kV/cm. **(d)** Weibull statistical analysis of the breakdown field. **(e)** Unipolar P-E loops and **(f)** Energy storage performance as a function of temperature.

In addition, we have shown the endurance performance of the capacitors in the new Fig. S2(b). The response is stable in terms of ESD and efficiency up to 2×10^8 cycles.

This is included in the manuscript on page no. 17.

The charge-discharge response was also evaluated from transient current-time (I-t) measurements performed using square voltage pulses. The discharged energy density was calculated from the time-integrated Joule energy dissipated through the discharge resistance, using:

$$W_{dis}(t) = \frac{R}{V}\int_0^t I(t)^2\, dt$$

where R is the total discharge resistance (R = 100 Ω), I(t) is the measured time-dependent discharge current, and V is the active volume of the capacitor. The resulting $W_{dis}(t)$ curves, shown in Fig. S2(c), exhibit a rapid increase followed by a well-defined saturation plateau. The plateau value corresponds to the discharged energy density at each applied field. As expected, the W_{dis} plateau increases with increasing nominal electric field, confirming that more energy is released from the capacitor when it is charged to higher fields. At the highest applied field of 1100 kV/cm, we obtain a W_{dis} value of 16.3 J/cm^3, which is close to the ESD value of 16.7 J/cm^3 obtained from the P-E loop. The minimal difference is due to contact resistance and internal serial resistances within the film, including grain boundaries and interfaces, which can dissipate part of the stored energy. (see: Communications Materials 6, 250 (2025)).
Figure S2(e) and (f) are new figures which illustrate the temperature-dependent unipolar P-E loops and the energy storage performance for the BCZT/STO SL. The response is stable in the measured temperature range. However, it is possible to observe that the loops start to become linear after 85ºC, which is close to T_m, the temperature of maximum permittivity, observed in the dielectric measurements. Nevertheless, the BCZT/STO SL shows stable energy storage performance up to 110 ºC, with no obvious performance degradation.
This is included in the manuscript on pages no. 18 and 19.

These additional measurements (frequency stability, temperature stability, endurance and charge-discharge testing) provided in Fig. S2 strengthen the conclusion that the BCZT/STO superlattice design enables a stable, highly efficient energy storage response at moderate applied electric fields.

Reviewer #2:

This manuscript presents an innovative approach to enhancing energy storage performance in lead-free relaxor ferroelectric capacitors through superlattice (SL) engineering, combining BCZT and STO layers with asymmetric electrodes (LSMO/Au). However, the manuscript suffers from several issues that undermine the strength of its claims as follows:

We thank the Reviewer for the detailed and constructive comments. We have revised the manuscript to address the concerns raised regarding the strength of our claims and provide additional supporting data and analysis below.

1. The manuscript reports ESD = 12.6 J/cm³ at 920 kV/cm and 20.4 J/cm³ at 1280 kV/cm for the BCZT/STO SL. However, these values do not surpass recently published results in lead-free systems such as Nat. Commun. 16, 886 (2025) and Nat. Commun. 16, 1300 (2025). The manuscript's primary performance advantage appears to be the ultra-high efficiency (η ~ 99%) rather than ESD. However, this extreme efficiency value is highly sensitive to measurement uncertainty.

A: The two cited works report ESD values of [12.7 J/cm³ at 700 kV/cm, η = 87.2%] and [22.0 J/cm³ at 750 kV/cm, η = 96.1%], respectively, which do indeed exceed the ESD reported here. However, these values are achieved in micrometer thick samples at breakdown voltages roughly 40× higher than the maximum voltage used in this work. Our central claim is not the highest absolute ESD, but the **combination of high ESD at a low-to-moderate field/voltages with ultra-high efficiency (η ≈ 99%), which is the relevant figure of merit for low-voltage, miniaturized applications.**

For instance, relaxor ferroelectric thin films at moderate applied electric fields (~1000 kV/cm) are crucial for developing high-power, miniaturized pulse-power electronic systems, such as advanced capacitors, micro-electromechanical systems (MEMS), and high-temperature actuators. They provide large recoverable energy density and high efficiency, allowing for rapid charge-discharge speeds. Ceramics based on relaxor ferroelectrics, on the other hand, cannot be used for the same purpose given the fact that they need a much higher voltage to get a similar performance given the fact that they are much thicker. For example, both ceramics and polymers often have to be operated at a high applied voltage (1–10 kV) due to their large thickness (few tens of micrometers to millimeters), which limits the range of practical applications (see: Energy Storage

Materials Volume 25, March 2020, Pages 193-201). Therefore, we agree with the Reviewer regarding that the advantage is the ultra-high efficiency ($\eta \sim 99\%$).

2. The manuscript repeatedly emphasizes "low applied electric field" as a key advantage. Therefore, the authors should provide a clear quantitative definition of what constitutes a "low electric field" in the context of thin film capacitors, with reference to the practical voltage requirements of target applications. Furthermore, the ESD normalized by electric field should be compared with other relevant reported references.

A: We thank the Reviewer for asking us to clarify this. In the context of thin-film capacitors, we define "low electric field" operation as ≤ 2.5 MV/cm, as opposed to the >3 MV/cm fields typically required to reach the highest reported ESD values in perovskite RFE/FE thin films. For a film thickness of 200 nm as used here, this corresponds to an applied voltage below 50 V, consistent with the supply voltages of typical miniaturized/wearable electronics (See: Energy Storage Materials Volume 25, March 2020, Pages 193-201).

For a low voltage range, typically < 50 V, relaxor ferroelectrics are used for high-density, ultra-fast energy storage in miniaturized electronics (see: Energy Storage Materials Volume 25, March 2020, Pages 193-201). For instance, in wearable and implantable devices, space is the primary constraint and for that reason thin films, instead of ceramic-based capacitors, are required.

This is included in the manuscript on pages no. 3 and 4.

Following the Reviewer's suggestion, we have also added a new benchmark comparison using the recoverable energy storage density normalized by the applied electric field:

$$U_e = \frac{ESD}{E}$$

As shown in Fig. 5(c), the BCZT/STO SL exhibits a competitive performance within the low-to-moderate field range considered. Although the SL does not show the highest U_e among all reported lead-free thin film capacitors, it combines a competitive U_e with a significantly higher efficiency than most comparable lead-free ferroelectric and relaxor ferroelectric thin-film systems.

This is included in the manuscript on pages no. 19 and 20.

Therefore, **the main advantage of the BCZT/STO superlattice** is not solely the maximization of U_e, but the **simultaneous achievement of recoverable energy storage under low absolute operating voltage and ultra-high efficiency.** This additional comparison has been included in the revised manuscript to provide a more quantitative and balanced assessment of the low-field performance.

3. The manuscript attributes the observed imprint effect in the P-E hysteresis loops entirely to the work function difference between the LSMO bottom electrode and the Au top electrode. However, imprint in ferroelectric thin films has multiple possible origins. The manuscript provides no control experiments to isolate the electrode asymmetry effect, nor does it present leakage current or interface charge distribution measurements to verify the work function hypothesis.

A: We thank the Reviewer for raising this point. We agree that to definitely attribute the imprint effect to the electrode work-function contribution would require a symmetric-electrode control sample and leakage-current/interface-charge measurements. However, the strain and compositional data in Fig. S3, together with the disappearance of imprint in the thin-layer (~3 nm) BCZT/STO SL (Fig. S5), indicates that substrate induced strain gradient is the dominant effect. We have revised the manuscript text to state this as the dominant origin of the imprint effect, and we note the electrode contribution as a negligible effect.

This is included in the manuscript on pages no. 10 and 11.

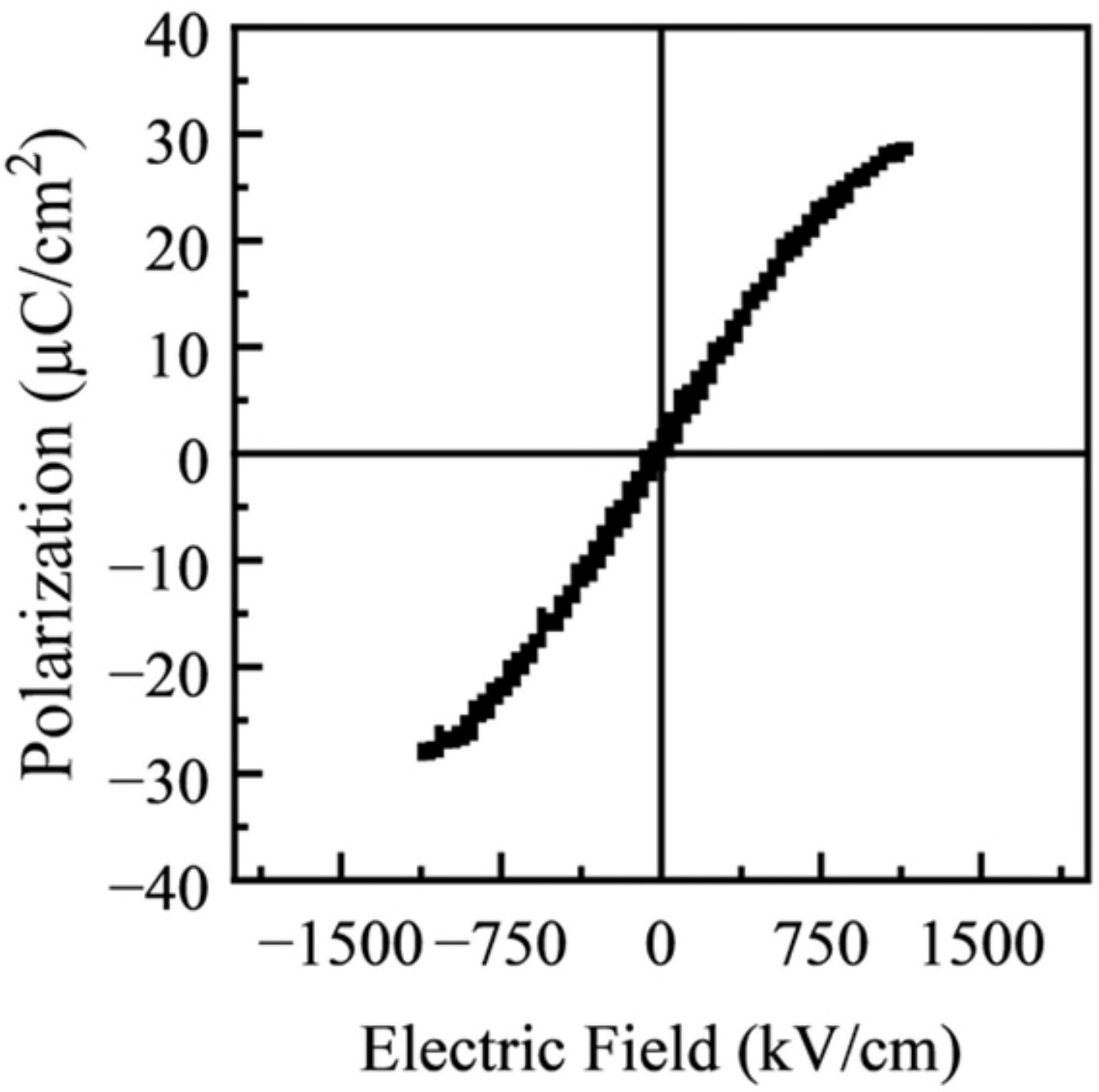

Figure S5. Polarization-electric field (P-E) hysteresis loops for a BCZT/STO SL with each individual layer has a thickness of ~3 nm.

4. The figure of merit Uf = ESD/(1 − η) is numerically unstable near η → 100%, and the claimed "5× higher" comparison is unreliable. The authors should explicitly discuss the sensitivity of Uf to η measurement uncertainty and provide error propagation analysis.

A: We thank the Reviewer for this important and constructive comment. We agree that the figure of merit $U_f = ESD/(1 - \eta)$ becomes highly sensitive to small variations in efficiency when η approaches 100%. We have considered the errors in ESD and η to be 1.97 J/cm^3 and 2.6%, respectively, taking into account the evaluation of the energy storage performance recorded for 20 different measurements. The uncertainty in U_f was estimated and the U_f is equal to $1723 \pm 273\ J/cm^3$, which means that the error in U_f is around 16%. This calculation confirms that the central value of U_f reported in the table is correctly calculated from the measured ESD and the corresponding efficiency. Overall, the **reported U_F is by far the highest value reported in the literature for representative lead-free FE and RFE thin films**.

This is included in the manuscript on page no. 19.

5. The manuscript claims that "a higher breakdown electric field is obtained in the BCZT/STO SL compared to the BCZT film" but provides no Weibull statistical analysis and no information on the number of samples tested for breakdown.

A: We thank the Reviewer for this important suggestion. A Weibull statistical analysis of the breakdown field for the BCZT/STO SL is now given in Fig. S2(d). Breakdown measurements were performed on n = 10 BCZT/STO SL capacitors and a breakdown field value of $E_{BD} = 1342 \pm 12$ kV/cm and a Weibull modulus of $\beta = 18.8 \pm 2.9$ were achieved. The uncertainty in E_{BD} was calculated by propagating the uncertainty of the linear fit parameters, including the covariance between the intercept and slope. The relatively high Weibull modulus indicates a narrow breakdown distribution and excellent capacitor reproducibility for the BCZT/STO SL. (see: Scientific Reports volume 15, Article number: 21617 (2025) and Nature Communications volume 9, Article number: 1813 (2018)).

This is included in the manuscript on page no. 19.

6. The manuscript's central claim is supported only by static HAADF-STEM observations. This is insufficient to establish a causal relationship between the SL structure and the observed polar nanoregion configuration. Theoretical calculations such as phase-field simulations are necessary to supplement the experimental observations and analyze the relationship between SL structure and polar nanodomain formation.

A: We agree that phase-field simulations would provide additional mechanistic insight. Performing new simulations specifically for the BCZT/STO system is beyond the scope of the present revision. However, prior phase-field work on related $SrTiO_3$-based superlattices ($BaTiO_3/SrTiO_3$, $BiFeO_3/SrTiO_3$ [Nanomaterials 2026, 16(10), 582 and Advanced Materials 34, 2108772 (2022)]) demonstrates that strain engineering at layer interfaces controls polar-nanoregion/domain topology in this same class of materials, which supports the mechanism we propose. In order to not overlap the work, we have cited these works in the introduction as follows:

"Phase field simulations demonstrated the relevance of $BaTiO_3/SrTiO_3$ and $BiFeO_3/SrTiO_3$ superlattices where strain engineering is important to tune the polar topologies and enhance the energy storage performance".

This is included in the manuscript on page no. 4.

Moreover, we would like to mention that the causal relationship between the SL structure and the observed polar nanoregion configuration is not only demonstrated by HAADF-STEM images. Dielectric measurements in the SL and in the BCZT thin films capacitors clearly demonstrate that the relaxor behavior, commonly ascribed by the larger presence on polar nanoregions, is enhanced in the SL. In addition, the nanoscale polarization configuration leads to slimmer P-E hysteresis loops in the SL, which confirms that the polar nanoregion configuration has a significant impact in the dielectric, ferroelectric and energy storage properties.

Response Letter

Dear Editor,

Thank you very much for expediting the review of our manuscript titled “Superlattice Engineering in Relaxor Ferroelectric-based Capacitors for Ultra-Highly Efficient Recoverable Energy Storage Density" (Manuscript Ref.: NCOMMS-26-027279A). We appreciate the comments and suggestions from the Reviewers and are thankful to them for their invaluable time and effort in evaluating our work. As per the Reviewers’ comments, we have revised the manuscript incorporating all their suggestions. The modifications to the manuscript are highlighted in blue color font in the revised file. Specific responses to the Reviewer’s comments are listed below.

Please do let us know if any additional information is needed. Thank you.

Sincerely,

Dr. José Silva
University of Minho

Reviewer comments and author responses:

Reviewer #1:

The comments have been well addressed. It can be considered for publication in Nature Communications.

We thank the Reviewer for the critical reading of our manuscript and the positive feedback.

Reviewer #2:

Although the have responded constructively to several concerns, the most critical issue remain inadequately addressed. The original concern was that static HAADF-STEM images cannot establish a causal mechanism linking the superlattice structure to polar nanoregion behavior and energy-storage performance. The requested phase-field simulation (or equivalent GLD-based modeling) was not performed, with the authors stating it is “beyond the scope of this revision”.

The phase-field studies cited (BTO/STO, BFO/STO) concern polar topological structures, not polar nanoregions. Without theoretical modeling, the paper’s central claim that SL governs polar nanoregions formation remains unsubstantiated.

A: We thank the reviewer for their careful review and insightful comment. We fully agree that phase-field simulations are important for predicting or explaining emergent behavior in superlattices, such as polar topologies, like the ones reported in BTO/STO and BFO/STO (the cited works). Therefore, we would like to clarify that while phase-field simulations can predict the existence of polar nanoregions (PNRs) (See: Nat. Commun. 16, 886 (2025) and Nat. Commun. 16, 1300 (2025)), it is well known that BCZT thin films are relaxor ferroelectrics (RFE) containining PNRs, as already widely reported (See: https://doi.org/10.1063/5.0203962, https://doi.org/10.1063/1.1849817, https://doi.org/10.1016/j.jallcom.2026.187991, https://doi.org/10.1103/PhysRevMaterials.5.064415 and https://journals.aps.org/prb/abstract/10.1103/fhdr-wn22).

Here, we are not claiming that the superlattice governs the PNRs formation. Instead, we have combined a RFE which contains PNRs, with PE layers in a multilayered device that is promising for ES applications. In these RFE/PE SL structures, we anticipate that we PNRs from growing larger by SL confinement.

This is included in the manuscript on pages 3 and 4.

In fact, when an artificial periodic boundary condition is imposed to mimic a superlattice architecture, the phase-field framework restricts the spatial propagation of local random fields. This boundary constraint provides mathematical proof that the correlation length—and consequently the average radius of the PNRs—is strictly truncated in comparison to an unconstrained, monolithic relaxor counterpart.

To experimentally confirm the above, we performed additional work. Thus, we measured atomic-displacements from atomic-resolution HAADF-STEM images acquired from a BCZT thin film. The cross-sectional STEM image in Fig. S6(a) shows the BCZT/LSMO/STO heterostructure, while the atomic-resolution HAADF-STEM image in Fig. S6(b), acquired from the region indicated in Fig. S6(a), resolves the BCZT lattice at the unit-cell level. The B-site displacement analysis was performed using the same procedure employed for the BCZT layers in the SL. As shown in Fig. S6(c), the BCZT film exhibits spatially extended regions in which neighboring B-site displacement vectors retain a common orientation over considerably larger distances than those observed in the BCZT/STO SL. For clarity, vectors of uniform display length are used in this panel so that the spatial variation in orientation can be visualized independently of the displacement magnitude. The corresponding magnitude map is presented separately in Fig. S6(d). Importantly, the displacement-magnitude distribution in the BCZT film is

comparable to that obtained for the SL, with a median displacement of approximately 30 pm. Thus, the principal difference between the two systems is not the magnitude of the local polar displacement, but its spatial organization. Consistent with this observation, the angular distribution of the BCZT film is strongly concentrated around the dominant ~90° orientation, whereas a substantially broader distribution of local polar orientations is observed in the BCZT/STO superlattice. These observations demonstrate that nanoscale polar displacements are already present in the BCZT film and support a more spatially correlated polar state in the single-layer film, whereas the SL exhibits a more fragmented polar configuration. Thus, our results indicate that SL engineering reduces the characteristic spatial correlation of the pre-existing PNRs in BCZT, consistent with confinement imposed by the periodic BCZT/STO interfaces.

The coexistence of smaller PNRs observed in BCZT/STO SL therefore provide valuable direct microscopic evidence for the smaller characteristic length of polar domains in SL compared with the single film that is highly favorable for high ŋ ES capacitors.

This is included in the manuscript on pages 14 and 15, and also in page 20.

We have also slightly changed the abstract and the introduction in order to further clarify this important remark made by the Reviewer.

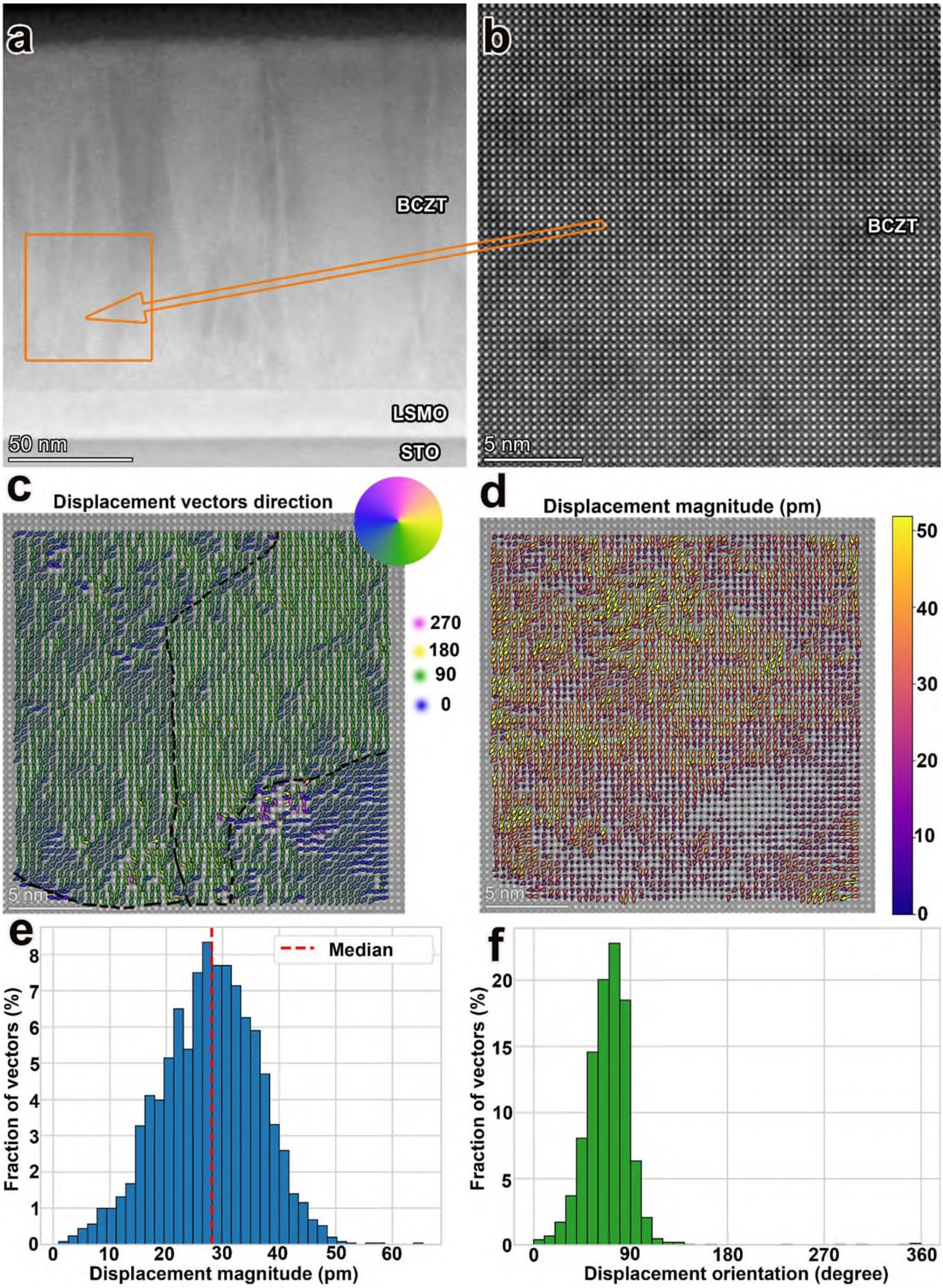

Figure S6. Displacement-vector mapping of the BCZT/LSMO/STO heterostructure. **(a)** Atomic-resolution HAADF-STEM overview image of the BCZT/LSMO/STO

heterostructure viewed along the [100] zone axis. **(b)** Magnified view of the BCZT area outlined by the orange square. **(c)** Displacement-vector map colored by vector orientation. **(d)** Displacement-vector map colored by displacement magnitude; black arrows serve as visual guides for the larger PNR regions. **(e)** Polarization-magnitude distribution extracted from the analyzed PZT region. **(f)** Angular distribution of the polarization vectors.

Response Letter

Dear Editor,

Thank you very much for expediting the review of our manuscript titled "Superlattice Engineering in Relaxor Ferroelectric-based Capacitors for Ultra-Highly Efficient Recoverable Energy Storage Density" (Manuscript Ref.: NCOMMS-26-027279B). We appreciate the comments and suggestions from the Reviewers and are thankful to them for their invaluable time and effort in evaluating our work. Specific responses to the Reviewer's comments are listed below.

Please do let us know if any additional information is needed. Thank you.

Sincerely,
Dr. José Silva
University of Minho

Reviewer comments and author responses:

Reviewer #2:

The authors have thoroughly addressed the comments. Accordingly, I recommend this paper for acceptance.

We thank the Reviewer for the critical reading of our manuscript and the positive feedback.
